# CDK4/6 Inhibitors for Breast Cancer Therapy—A Review of Clinical Trials, Structural and Computational Approaches

**DOI:** 10.3390/ph19040610

**Published:** 2026-04-10

**Authors:** Adela Avdičević, Samo Lešnik, Urban Bren, Luka Čavka

**Affiliations:** 1 Laboratory of Physical and Chemistry and Chemical Thermodynamics, Faculty of Chemistry and Chemical Engineering, University of Maribor, Smetanovaulica 17, SI-2000 Maribor, Slovenia; adela.avdicevic@um.si (A.A.); samo.lesnik@um.si (S.L.); urban.bren@um.si (U.B.); 2 Institute of Environmental Protection and Sensors, Beloruska ulica 7, SI-2000 Maribor, Slovenia; 3 Faculty of Mathematics, Natural Sciences and Information Technologies, University of Primorska, Glagoljaška ulica 8, SI-6000 Koper, Slovenia; 4 Department of Oncology, University Medical Centre Maribor, Ljubljanska cesta 5, SI-2000 Maribor, Slovenia

**Keywords:** CDK4/6, abemaciclib, palbociclib, ribociclib, molecular dynamics simulations, drug resistance

## Abstract

Cyclin-dependent kinases 4 and 6 (CDK4/6) play a central role in the regulation of cell cycle progression and represent important therapeutic targets in hormone receptor-positive, human epidermal growth factor receptor 2-negative (HR+/HER2−) breast cancer. The introduction of selective CDK4/6 inhibitors, including palbociclib, ribociclib, and abemaciclib, in combination with endocrine therapy, has significantly improved clinical outcomes and has become a standard treatment strategy in both metastatic and high-risk early-stage disease. Nevertheless, treatment resistance and disease progression remain major clinical challenges. A deeper understanding of the structural characteristics of CDK4/6 and the molecular basis of inhibitor binding is therefore essential for improving therapeutic strategies and guiding the development of new targeted agents. This review provides an integrated overview of the structural features of CDK4/6 and their role in cell cycle regulation, summarizes the clinical development and major clinical trials of currently approved CDK4/6 inhibitors, and discusses recent computational studies investigating inhibitor binding and conformational dynamics. Particular attention is given to the application of in silico approaches, including molecular docking, molecular dynamics simulations, and binding free-energy calculations, which provide insights into mechanisms of therapy resistance and potential strategies to overcome them and support the identification and optimization of novel CDK4/6-targeted therapeutic candidates. By integrating structural, clinical, and computational perspectives, this review highlights current knowledge and emerging directions in CDK4/6 research that may advance the development of more personalized therapies for HR+/HER2− breast cancer, while accounting for both intrinsic and de novo resistance mechanisms.

## 1. Introduction

Breast cancer (BC) is the most commonly diagnosed cancer in women worldwide and the second most commonly diagnosed cancer overall, with an estimated 2.3 million new cases in 2022, accounting for 11.6% of all newly diagnosed cancers globally, and approximately 670,000 deaths worldwide [1]. It is a heterogeneous disease with distinct subtypes that differ in their biological characteristics, disease stage (localized, locally advanced, or metastatic), response to treatment, and survival outcomes [2]. The most common biological subtype of breast cancer is hormone receptor-positive (HR+) disease, characterized by estrogen-driven growth mediated through the estrogen receptor (ER), and it accounts for approximately 60–70% of all BC cases [3,4]. The cornerstone of HR+ BC treatment represents endocrine therapy (ET), which is often combined with cyclin-dependent kinase 4/6 (CDK4/6) inhibitors, both in the metastatic setting and in high-risk patients following surgery and adjuvant chemotherapy with or without postoperative irradiation [5,6].

Most commonly, BC is diagnosed in the early stage and is curable in around 75% of cases [4]. In the developed world, about 5–10% of patients are diagnosed with metastatic disease at presentation [7,8]. Despite curative-intent locoregional therapy for early-stage BC, approximately 12–23% of patients will eventually develop advanced BC (ABC), sometimes decades after the initial treatment [9]. Both primary metastatic and recurrent ABC remain incurable. However, novel therapies have significantly prolonged survival and improved quality of life for many patients [10,11]. In contrast, for early-stage disease with high-risk features (such as lymph node involvement, larger tumor size, or high grade), the goal is to reduce the risk of recurrence through adjuvant systemic therapies. In HR+ early-stage breast cancer, these typically include chemotherapy and ET, and, recently, CDK4/6 inhibitors have been introduced into clinical practice [4,12]. In addition, natural-based therapies are being explored for an auxiliary supportive role in BC management [13,14]. For example, prophylactic use of EGCG solution significantly reduced the incidence and severity of radiation-induced dermatitis in patients receiving adjuvant radiotherapy for BC [15]. From another perspective, wild strawberry (*Fragaria vesca* L.) has attracted interest due to its antibacterial, antioxidant, antitumor, and anticancer properties and has been investigated in MCF-7 cell lines [16].

Over the past decade, preclinical and clinical research have focused on the development of new therapeutic approaches in HR+ breast cancer aimed at prolonging or restoring endocrine sensitivity, reducing the need for chemotherapy, and improving patients’ quality of life [4,17]. CDK4/6 inhibitors play a key role in delaying the development of endocrine resistance by regulating cell cycle progression, particularly the transition between the G1 and S phases of cell division [18,19,20], when DNA synthesis and proper chromosome segregation take place [18,19]. In addition to regulating the cell cycle, CDKs are also involved in numerous other biological processes, including apoptosis [21], transcription [22], epigenetics [23], angiogenesis [24], hematopoiesis [25], metabolism [26], and DNA repair [27,28].

CDK4/6 inhibitors such as palbociclib, ribociclib, and abemaciclib have emerged as an important targeted therapy in recent years [4,29,30]. Originally approved for use in the metastatic setting of HR-positive breast cancer, CDK4/6 inhibitors have recently gained broader adoption in high-risk early-stage disease, aiming to lower the likelihood of recurrence [31].

According to internationally accepted guidelines, CDK4/6 inhibitors are always used in combination with ET (e.g., aromatase inhibitors, ovarian function suppression, or antagonists of ER) in both the metastatic setting and early stages [32,33,34]. In patients with metastatic disease, this combination significantly prolongs progression-free survival (PFS) compared to ET alone; however, overall survival (OS) benefit was proven only for ribociclib [4,30,35,36]. Overall, the toxicity profiles of all three agents are well characterized and can be effectively managed in clinical practice [37].

Despite the substantial benefit of CDK4/6 inhibitors in the treatment of metastatic disease, a considerable proportion of patients experience disease progression within two years. In pivotal phase III trials (PALOMA, MONALEESA, and MONARCH), a median progression-free survival of approximately 24–28 months indicated that nearly half of patients progress within this time-frame [36,38,39]. This underlines the need for further research and innovative strategies to prevent or overcome resistance and thus improve long-term treatment outcomes [40,41]. Although all three CDK4/6 inhibitors are approved for use in the metastatic setting, there is no evidence that personalized selection improves clinical outcomes. Given inter-patient variability in CDK4/6 structure, it would be reasonable to investigate whether tailored matching of specific CDK4/6 inhibitors to individual structural enzymatic profiles could provide additional benefit.

To address these limitations and support the development of improved therapeutic strategies, this review provides an integrated overview of CDK4/6 structure, role, inhibition and resistance in HR+/HER2− breast cancer. It combines functional and structural insights with clinical evidence and computational approaches to present a comprehensive understanding of current treatment options and ongoing research efforts. The first part of the review outlines the role of CDK4/6 in cell cycle regulation and highlights key structural features relevant to kinase activation and inhibitor binding. This is followed by a discussion of approved CDK4/6 inhibitors, including their pharmacological properties and findings from clinical studies. The final section focuses on recent advances in the application of in silico methods for the identification and optimization of novel CDK4/6 inhibitors of both natural and synthetic origin.

## 2. CDK4/6 Roles in the Cell Cycle

Cell proliferation represents one of the fundamental biological processes in living organisms that involves cell growth, replication, and division [12]. These phases together form the cell cycle, the progression of which is also regulated by cyclins, CDKs, and their inhibitors [42].

**Figure 1:**
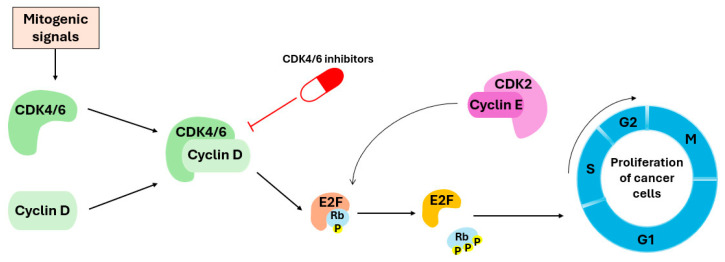
Mitogenic signals promote the assembly and activation of the cyclin D–CDK4/6 complex, which phosphorylates the retinoblastoma protein (pRb). This phosphorylation releases the transcription factor E2F, enabling the transition from the G1 to the S phase, where DNA replication begins. CDK4/6 inhibitors block activation of this complex, prevent pRb phosphorylation, and consequently halt cell cycle progression. Because the activity of CDK4/6 inhibitors requires functional pRb protein, their therapeutic benefit is largely confined to tumors with preserved pRb, whereas many other cancer types exhibit pRb loss and are therefore unresponsive [47].

Most differentiated cells in an adult organism reside in the G0 phase, where they remain dormant until they receive a mitogenic signal (Figure 1) [43,44]. After receiving the signal, cyclin–CDK complexes get activated, and subsequently tightly regulate cell cycle transitions [45,46].

In the early phase of the cell cycle, CDK4 and CDK6 are activated by binding to one of the three isoforms of cyclin D (D1, D2, or D3), forming a functional complex. This complex initiates a cascade of biochemical processes that allow cells to move from the pre-replication phase (G1) to the replication phase (S), which represents one of the most critical checkpoints of the cell cycle (Figure 1) [19,48,49]. One of the key functions of CDK4/6–cyclin D complex is to phosphorylate the tumor suppressor pRb and its related pocket proteins p107 and p130 [50,51,52].

In late G1, cyclin D–CDK4/6 activity gradually declines, while cyclin E levels rise. This promotes formation of the cyclin E–CDK2 complex, which further phosphorylates pRb and drives S-phase entry, until cyclin E is ultimately degraded in the proteasome [53]. Later, cyclin E is replaced by cyclin A (in the S phase), which, in complex with CDK2, continues to phosphorylate the proteins responsible for DNA replication [54].

The regulation of CDK4/6 is tightly controlled throughout the cell cycle, with inhibitory mechanisms playing a crucial role in preventing uncontrolled cell proliferation. Endogenic cyclin kinase inhibitors (CKIs) slow down or stop the CDK4/6 activity and thus regulate cell division. CKIs contain a kinase inhibitory domain (KID) at their N-terminus, which enables specific binding to individual cyclin/CDK complexes and inhibits their function [55]. The most important CKIs are divided into two families: (i) the INK4 family (p15, p16, p18, and p19), which specifically targets CDK4 and CDK6 and prevents their binding to D-type cyclins [56,57,58,59,60], and (ii) the CIP/KIP family (p21 (CIP1), p27 (KIP1), and p57 (KIP2)), which inhibits multiple CDKs [55].

In tumor cells, such cell cycle control mechanisms are often disrupted, leading to uncontrolled division. The deletion or inactivation of inhibitors from the INK4 family (such as p16INK4A) and mutations in the cell cycle repressors, such as RB1 and TP53, reduce control over CDK4/6 activity. P53 regulates CDK4/6 via the inhibitor p21 (CIP1); as such, its loss of function further promotes activity of the complex [19].

Alterations in the genes encoding key cell cycle regulators can compromise their inhibitory function on CDK4/6, thereby promoting aberrant cell cycle progression or the increased expression of cyclin D (especially cyclin D1), leading to an excessive activation of the cyclin D-CDK4/6 complex. Moreover, CDK4/6 mutations reduce their sensitivity to CKIs [61,62]. An increased expression of cyclin E can bypass CDK4/6 inhibition and activate CDK2 [63]. Overall, this results in the excessive phosphorylation of the Rb protein, compromising its tumor-suppressive function [64]. Since phosphorylated Rb can no longer inhibit the transcription factor E2F, the cells begin to proliferate uncontrollably [61,62]. They become independent of mitogenic signals and continue to progress through the cell cycle without external regulation, driving malignant transformation and facilitating metastatic processes [21,54,65].

Since excessive activation of CDK4/6 represents an important factor in the uncontrolled proliferation of tumor cells, these kinase complexes have become important targets in the development of targeted therapies. The CDK4/6 inhibitors used in clinical practice are orally administered drugs specifically designed to inhibit the activity of these kinases, thereby preventing phosphorylation of the pRb and preserving its tumor suppressor function. As a result, pRb remains bound to the transcription factor E2F, preventing the transition of the cell cycle to the S phase and inhibiting DNA synthesis. This, in turn, slows down or even halts tumor cell growth, making CDK4/6 inhibition an effective therapeutic approach for certain cancer types, including BC [35,66].

A comprehensive understanding of the regulation and inhibition of CDK4/6 requires an in-depth study of their structure, which determines the activity, cyclin-binding specificity and interactions with inhibitors. Computational studies play a central role in this process as they facilitate a high-resolution modeling of CDK4/6 inhibitor interactions, virtual screening of potential drug candidates, and the prediction of conformational changes upon ligand binding. In addition, these in silico approaches enable the investigation of the structural and functional consequences of mutations and provide atomistic insights into altered binding patterns and resistance mechanisms. These methods can significantly accelerate the rational design of selective and effective inhibitors [18,67,68,69].

## 3. CDK4/6 Structure

**Figure 2:**
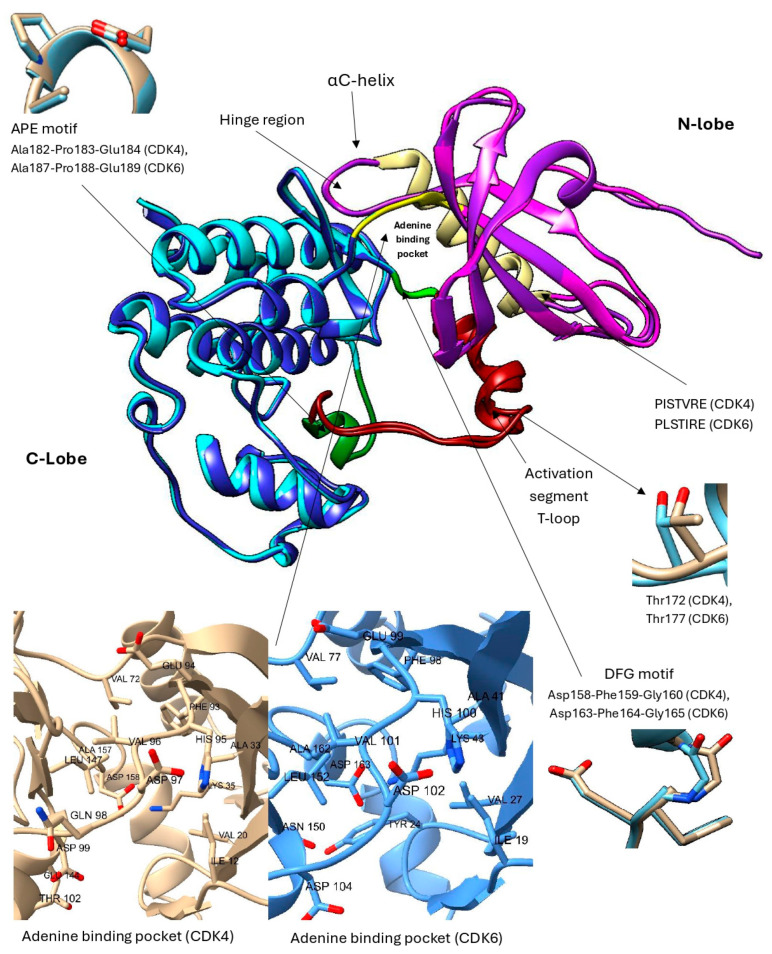
Presentation of the most important structural elements of superimposed CDK4/6. CDK4 consists of the N-lobe (pink) and the C-lobe (light blue), while in CDK6, the N-lobe is depicted in purple and the C-lobe in dark blue. The flexible hinge region is highlighted in yellow, the αC-helix in ochre, the DFG motif in light green, the APE motif in dark green and the activation segment in dark red. The inset displays the phosphorylation-sensitive residue Thr172 in CDK4 and Thr177 in CDK6, the APE motif (Ala182–Pro183–Glu184 in CDK4 and Ala187–Pro188–Glu189 in CDK6), the DFG motif (Asp158–Phe159–Gly160 in CDK4 and Asp163–Phe164–Gly165 in CDK6), and the adenine binding pockets [80,81,82].

The CDK4 and CDK6 proteins share 71% sequence similarity, which is also reflected in their related functions [66]. CDK4 consists of 303 amino acids, while CDK6 has 326 amino acids, both forming a characteristic bilobal structure (Figure 2) [18,21]. They are serine/threonine kinases that act as separate catalytic subunits forming complexes with D-type cyclins, rather than assembling into a shared CDK4/6 complex. Their structure includes the N-lobe (residues 1–96 in CDK4 and 1–100 in CDK6), which consists of five antiparallel β-sheets and an αC-helix in both CDKs. Within this domain, a conserved 16-amino acid motif (PISTVRE in CDK4 and PLSTIRE in CDK6) is critical for ATP binding [21,56,70]. The C-lobe spans residues 97–303 in CDK4 and 101–326 in CDK6 and consists predominantly of α-helical elements. It also contains the activation segment, known as a T-loop, which is critical for regulating kinase activity. In CDK4, the T-loop encompasses residues 163–189, whereas in CDK6 it spans residues 168–193 [18,21,71,72,73,74]. In CDK4 and CDK6, the activation segment is delineated by two conserved structural motifs, extending from the DFG motif (CDK4: 158–160; CDK6: 163–165) to the APE motif (CDK4: 182–184; CDK6: 187–189), which together frame the regulatory region essential for kinase activation [74]. A flexible hinge region (residues 93–96 in CDK4 and 99–102 in CDK6) connects the two lobes, allowing for conformational changes and forming hydrogen bonds with ATP to stabilize its binding (Figure 2) [71,72,73,74]. Structural differences in the hinge region confer greater conformational flexibility to CDK4, enabling it to adopt a wider range of active and inactive states. In contrast, the hinge region of CDK6 is more rigid, stabilizing the kinase predominantly in an inactive conformation [75].

Although cyclin D binding is required for activation, CDK4/6 does not instantly adopt an active conformation following this interaction. The αC-helix remains displaced, and the activation loop is either largely disordered or it adopts a conformation that occludes the active site, leaving the ATP-binding pocket inaccessible to substrates [70,76]. A full activation of the CDK4/6–cyclin D complex requires an additional phosphorylation within the T-loop by the CDK-activating kinase (CAK), which facilitates the structural rearrangements necessary for the substrate binding and enzymatic function [72]. CAK corresponds to the active trimeric complex of CDK7, cyclin H, and MAT1. Although it is present throughout the cell cycle, its catalytic activity depends on phosphorylation of CDK7 at Thr170 and on the integrity of the trimeric assembly, which fine-tunes its efficiency toward CDK4/6 [77,78]. The activation segment includes phosphorylation-sensitive residues, Thr172 in CDK4 and Thr177 in CDK6 [70] (Figure 2).

Interestingly, the length and orientation of the αC-helix represent the key determinants of cyclin D binding specificity. CDK4 exhibits a longer αC-helix that favors binding to cyclin D1, whereas CDK6 associates more firmly with cyclin D3. This specificity is mainly attained through electrostatic and hydrophobic interactions of the αC-helix [79].

Given the central role of ATP binding for kinase function, the ATP-binding site has emerged as an attractive target for pharmacological interventions. Selective CDK4/6 inhibitors, abemaciclib, palbociclib, and ribociclib, operate by competitively binding to the ATP-binding site. By occupying the ATP-binding pocket, these inhibitors actively prevent the phosphate transfer to the substrate, thereby effectively halting enzymatic activity [41].

## 4. CDK4/6 Inhibitors

Over the last 30 years, intense efforts to develop small-molecule inhibitors of cyclin-dependent kinases (CDKs) have yielded only modest success [83]. First- and second-generation compounds, of which more than 20 reached clinical trials, were broadly active and included first-generation inhibitors, such as flavopiridol and roscovitine, and second-generation inhibitors, such as dinaciclib, blocking not only the CDKs that drive cell cycle progression but also transcription-associated CDKs [84,85]. Earlier CDK inhibitors were less selective and associated with greater toxicity, whereas modern agents demonstrate improved tolerability with generally similar but lower-grade adverse effects [86,87]. As a result, the first- and second-generation CDK inhibitors have not found significant clinical applications in cancer treatment [83]. In contrast, third-generation inhibitors represent a more promising class, as they offer wider therapeutic windows [88].

**Figure 3:**
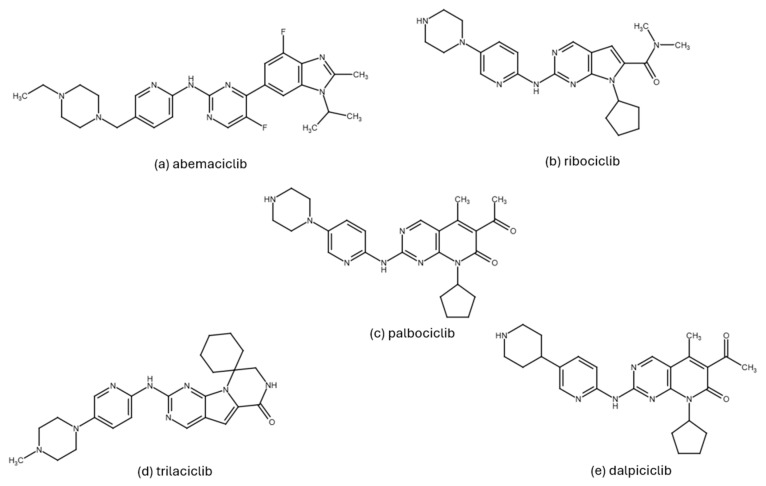
Chemical structures of the third-generation CDK4/6 inhibitors: abemaciclib (**a**), ribociclib (**b**), palbociclib (**c**), trilaciclib (**d**) and dalpiciclib (**e**).

To overcome the described limitations, the third-generation CDK inhibitors were designed with improved selectivity and safety profiles. Currently, three selective third-generation CDK4/6 inhibitors, palbociclib, ribociclib, and abemaciclib, are approved by regulatory agencies for the treatment of estrogen receptor-positive/human epidermal growth factor receptor 2-negative (ER+/HER2−) breast cancer [17,84,89]. Moreover, dalpiciclib has been approved by the Chinese FDA [37]. These represent ATP-competitive inhibitors with a high specificity for CDK4/6 and minimal effects on other CDKs, providing effective antitumor activity with acceptable toxicity profiles [40,58]. In addition to their similar structure (Figure 3) and binding mechanism [89], the drugs also have comparable pharmacokinetic properties [90]—all of them are predominantly metabolized by the cytochrome P450 enzyme, CYP3A4, and can be applied orally.

In addition to CDK4/6 inhibitors, which are primarily used to treat HR+/HER2− breast cancer, yet another CDK4/6 inhibitor with a different therapeutic target, trilaciclib, is also clinically relevant. In February 2021, it was approved by the FDA as an adjuvant therapy for patients with metastatic small-cell lung cancer. By protecting hematopoietic stem and progenitor cells from the chemotherapy-induced damage, trilaciclib effectively reduces the incidence of myelosuppression [17,58].

Each of the FDA-approved CDK4/6 inhibitors displays unique pharmacological properties that merit individual attention. Palbociclib (PD-0332991, Ibrance) represents a pyridopyrimidine derivative approved for the treatment of patients with advanced or metastatic HR+/HER2− breast cancer. It is highly potent, with IC_50_ values of 11 nM for CDK4 and 16 nM for CDK6 [29,58,91]. The standard dosing regimen is 125 mg daily for three weeks followed by a one-week break. After oral administration, the maximum plasma concentration (Cmax) is reached within 6–12 h, while the elimination half-life ranges from 24 to 34 h [92].

Ribociclib (Kisqali, LEE011) works via a similar mechanism to palbociclib and exhibits a structurally related scaffold. It also targets CDK4 (IC_50_ = 10 nM) and CDK6 (IC_50_ = 39 nM) [49]. It received approval for both metastatic and high-risk early HR+/HER2− breast cancer, and in combination with ET is administered on a 21-day-on/7-day-off dosing schedule; the standard dose is 600 mg and 400 mg for metastatic and adjuvant settings, respectively. Ribociclib reaches Cmax faster (within 1–4 h) and exhibits a slightly longer half-life of 30–55 h [58,92,93].

The third compound, abemaciclib (Verzenios, LY2835219), is indicated for both metastatic and early disease BC, always in combination with ET [48,74,94]. In preclinical studies, abemaciclib showed a strong inhibitory activity against CDK4 (IC_50_ = 2 nM) and CDK6 (IC_50_ = 9.9 nM), displaying an approximately five-fold greater selectivity towards CDK4. This greater activity toward CDK4 than CDK6 may be considered in light of the structural differences described above, including the greater conformational flexibility of CDK4, the comparatively more rigid conformation of CDK6 [75]. This selectivity contributes to the lower hematologic toxicity compared to the remaining two inhibitors. Moreover, abemaciclib also inhibits CDK9 (IC_50_ = 57 nM), and to a lesser extent CDK1, CDK2, and CDK7 [4,17,30,92]. It is typically administered twice daily in a dose of 150 mg, with Cmax reached in approximately 8 h and a half-life between 17 and 38 h [92]. An important advantage of abemaciclib emerges in the context of resistance. In certain in vitro models, cells resistant to palbociclib and ribociclib still respond to abemaciclib [66]. Additionally, its greater lipophilicity (cLogP 5.5 vs. 2.7 for palbociclib and 2.3 for ribociclib) enables broader tissue penetration, including potential blood–brain barrier crossing, and its reduced myelotoxicity profile may provide clinically meaningful advantages relative to the other CDK4/6 inhibitors [95,96,97,98]. In line with the structural observations discussed below, abemaciclib has been proposed to facilitate deeper burial within the ATP cleft, where it also displays a distinct local interaction pattern involving Lys43, His100 [51].

To better understand the molecular underpinnings of these differences, structural insights from crystallographic studies provide a valuable context. As of writing, the Research Collaboratory for Structural Bioinformatics Protein Data Bank (RCSB PDB) website provides access to 20 structures of CDK6 and 15 structures of CDK4 (Table 1). The structures discussed here were determined predominantly by X-ray crystallography, with the exception of 5FWK, 5FWL, 5FWM, and 5FWP [99], which are cryo-EM structures of CDK4 in complex with Hsp90 and Cdc37. However, the cryo-EM structures capture a chaperone-associated state rather than the canonical inhibitor- or cyclin-bound kinase structures considered here. 

Most of these structures are present in complexes with cyclins [100,101,102,103]; however, none of them cover the entire sequence. The lack of complete structures is due to the low electron density in the T-loop region, thereby making it difficult to capture this region by crystallographic methods [104]. Due to its flexibility and dynamic nature, the T-loop often does not adopt a stable conformation, making a precise visualization of this region in electron density maps difficult [105]. Available inhibitor-bound complexes include CDK6 structures with ribociclib (PDB ID: 5L2T), abemaciclib (5L2S), and palbociclib (PDB ID: 5L2I) [51], while for CDK4, only the abemaciclib complex (7SJ3) [106] is currently deposited.

**Table 1:** Structures of CDK4 and CDK6 from the RCSB PDB, with identifiers and descriptions of the complexes with different cyclins or inhibitors. With the exception of structures 5FWK, 5FWL, 5FWM, and 5FWP [99], which were obtained by cryo-EM and represent CDK4 in complex with Hsp90 and Cdc37, all currently available structures were determined by X-ray crystallography.

CDK4		CDK6	
PDB ID	Complex	PDB ID	Complex
3G33 [100]	CDK4–cyclin D3	1JOW [103]	CDK6–viral cyclin
7SJ3 [106]	CDK4–cyclin D3–abemaciclib	8I0M [107]	CDK6–inhibitor
2W99 [101]	CDK4–cyclin D	3NUP [108]	CDK6–inhibitor
2W9F [101]	CDK4–cyclin D	3NUX [108]	CDK6–inhibitor
2W9Z [101]	CDK4–cyclin D	4EZ5 [109]	CDK6–inhibitor
2W96 [101]	CDK4–cyclin D	6OQL [110]	CDK6–Cpd13
5FWK [99]	Hsp90–Cdc37–CDK4	6OQO [110]	CDK6–Cpd24
5FWL [99]	Hsp90–Cdc37–CDK4	1BI8 [111]	CDK6–p19^INK4D^
5FWM [99]	Hsp90–Cdc37–CDK4	2EUF [112]	CDK6–viral cyclin–palbociclib
5FWP [99]	Hsp90–Cdc37–CDK4	4TTH [113]	CDK6–viral cyclin–inhibitor
6P8E [102]	CDK4–Cyclin D1–P27	4AUA [109]	CDK6–ligand
6P8F [102]	CDK4–Cyclin D1–P27	2L2I [114]	CDK6–palbociclib
6P8G [102]	CDK4–Cyclin D1–P27	5L2S [51]	CDK6–abemaciclib
6P8H [102]	CDK4–Cyclin D1–P27	5L2T [51]	CDK6–ribociclib
9CSK [115]	CDK4–Cyclin D1–atirmociclib	1BLX [116]	CDK6–p19^INK4D^
		2F2C [112]	CDK6-viral cyclin–aminopurvalanol
		1XO2 [117]	CDK6–fisetin
		1BI7 [111]	CDK6–p16^INK4A^
		1G3N [118]	p18^INK4C^–CDK6–K–cyclin
		9D8U [115]	CDK6–atirmociclib

**Figure 4:**
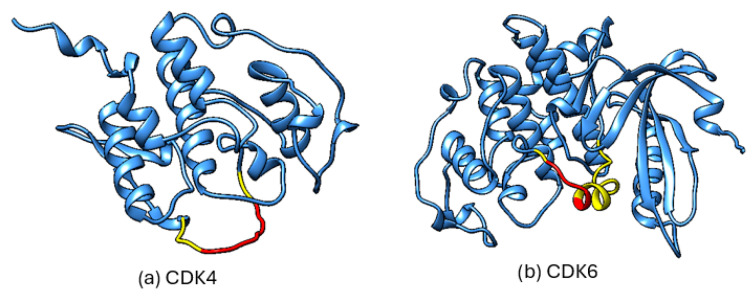
AlphaFold-predicted full-length structures of human CDK4 and CDK6 colored according to per-residue local model confidence (pLDDT). CDK4 is shown on the left, and CDK6 is on the right. Blue regions correspond to high-confidence parts of the models, whereas yellow to red regions indicate lower local confidence. Notably, the activation segment (T-loop-containing region), which is incompletely resolved in experimental CDK4 and CDK6 structures, also shows reduced confidence in the AlphaFold models [120].

In addition to the experimentally determined structures listed in Table 1, which often do not provide complete sequence coverage, the AlphaFold Protein Structure Database [119] offers predicted full-length models for human CDK4 and CDK6 that may serve as complementary structural resources. Of relevance is the activation segment (the T-loop-containing region), which is incompletely resolved in available crystal structures. However, as is shown in Figure 4, the corresponding region in the AlphaFold models displays reduced local confidence, indicating that these predictions should therefore be interpreted cautiously, as they represent uncertain models of a flexible region rather than reliably defined structures. In AlphaFold, local confidence is reported as pLDDT (predicted local distance difference test), a per-residue estimate of expected local structural accuracy based on the predicted agreement with experimentally observed local inter-residue distances [120].

The efficacy of CDK4/6 inhibitors has been confirmed in multiple clinical trials in which they consistently demonstrated an improved PFS in combination with standard ET only in patients with metastatic HR+/HER2− breast cancer. Despite their high efficacy, these therapies can be associated with adverse effects, so it is important to be aware of their frequency and severity. The most common toxicities are hematologic and gastrointestinal, with fatigue, nausea, neutropenia, and a lengthened heartbeat recovery time (prolonged QTc interval) being the most characteristic, while interstitial lung disease is less common [121]. Palbociclib and ribociclib are more frequently associated with neutropenia (70–80% of patients) [35], indicating a greater bone marrow toxicity and prolonged QTc interval, while abemaciclib more frequently causes gastrointestinal symptoms such as abdominal pain, diarrhea and taste changes. The incidence of fatigue, decreased appetite, and alopecia is similar for all three drugs [17,30]. More detailed results, including survival data, patient characteristics, and individual safety profiles, are summarized in Table 2, which provides a systematic overview of the main outcomes of studies that have shaped the current clinical practice.

Because the phase III trials of all three approved CDK4/6 inhibitors (PALOMA-2 and PALOMA-3; MONALEESA-2, -3, and -7; and MONARCH-2 and -3) were primarily designed and powered for progression-free survival (PFS), which served as the basis for regulatory approval and reimbursement in many countries, caution is warranted when portraying ribociclib as categorically superior solely on the basis of its consistent overall survival (OS) [139]. Ribociclib is the only agent that has demonstrated a statistically significant OS benefit in all three phase III registration trials. However, this should be interpreted in the context that OS was a secondary endpoint in these studies, and head-to-head phase III trials have yet to be done. Importantly, PALOMA-2 did not show a significant OS benefit, and MONARCH-3 showed a numerically favorable but not statistically significant OS improvement [36,133]. In contrast, the later-line endocrine-resistant trials PALOMA-3 and MONARCH-2 reported numerically prolonged OS in both of them [124,136]. At present, no validated biological explanation clearly accounts for why ribociclib showed a consistent OS advantage across trials, whereas palbociclib and abemaciclib did not [140]. Moreover, because all three agents are widely used in routine practice, real-world comparative analyses are particularly informative; so far, these studies have not demonstrated a clear OS advantage for ribociclib over the other CDK4/6 inhibitors [141]. Variability in OS outcomes across CDK4/6 inhibitor trials likely reflects differences in inclusion criteria, line of therapy, and statistical maturity. OS benefits are more readily detected in later-line settings, where higher event rates increase statistical power. In contrast, first-line trials are often limited by prolonged survival and a high proportion of censored observations, which may obscure potential OS differences. For instance, in PALOMA-2, the high degree of censoring and extended post-progression survival likely contributed to the lack of a statistically significant OS benefit [133].

The situation differs somewhat in the adjuvant setting, where phase III trials were primarily powered for invasive disease-free survival (iDFS). On this basis, only abemaciclib and ribociclib have received regulatory approval, although reimbursement varies considerably across countries. Several factors may contribute to the observed differences in efficacy, including longer treatment duration with ribociclib (3 years) compared with abemaciclib (2 years), as well as differences in dosing strategy, with continuous administration of abemaciclib versus the intermittent schedule used for ribociclib. In contrast to the metastatic setting, robust real-world outcome data for adjuvant CDK4/6 inhibition are currently lacking [128,134,138].

The SONIA trial has challenged the widely adopted paradigm in medical oncology that the most effective therapy should invariably be administered in the first-line setting of metastatic disease. Instead, its findings suggest that certain effective agents may retain their overall survival benefit when used sequentially in later lines, supporting a more flexible treatment sequencing strategy [127].

Despite the already established treatment options, the development of novel CDK4/6 inhibitors is still very active. At the time of the writing of this review, several other selective CDK4/6 inhibitors are still in clinical evaluation and play a key role in the development of targeted therapies for the treatment of metastatic HR+/HER2− breast cancer [49]. Table 3 provides a detailed overview of these inhibitors, including the individual agents, their developers, the stages of their corresponding clinical evaluation, and, where available, their reported IC_50_ values, thereby illustrating that these compounds remain at different points of development and characterization. The IC_50_ values for CDK4, CDK6 and for other targets such as CDK9 provide an important insight into the inhibitory potency and specificity of the inhibitors. Some inhibitors achieve IC_50_ values in the low nanomolar (nM) range, indicating a high inhibitory potential and potentially lower required doses, which could reduce the risk of toxicity [142].

The continued development of novel selective CDK4/6 inhibitors reflects the considerable need to overcome resistance, which has repeatedly emerged in clinical studies as a major limitation of current therapy.

## 5. Mechanisms of Resistance to CDK4/6 Inhibitors

Resistance to CDK4/6 inhibitors represents a spectrum from intrinsic to acquired mechanisms. Intrinsic resistance is characterized by a lack of response at treatment initiation, whereas acquired resistance emerges over time through tumor adaptation under selective pressure, resulting in clonal expansion of resistant cells (typically as a consequence of genetic mutations or activation of alternative signaling pathways) and eventual disease progression [151]. The most compelling explanation for intrinsic resistance is the tumor’s inherent independence from CDK4/6-mediated cell cycle control, thereby lacking a cell cycle regulatory machinery that can be exploited by these agents [152]. In such cases, CDK4/6 inhibitors lack a functional target through which to exert antitumor activity. A prototypical example is RB1 inactivation (see Figure 1), while other resistance mechanisms share biological features with hormone-independent (non-luminal) breast cancer [153].

Inefficient Rb play significant role in both intrinsic and acquired resistance; recently, a special event described as “double hit”, meaning “simultaneous inactivation of both alleles of the RB1”, has been identified, arising from complete gene deletions, deteriorating point mutations, or loss of heterozygosity (LOH) [47]. Therefore, in the future, there are some prospects in continuous assessment of Rb status (mutations or its expression) for optimizing the management of metastatic breast cancer. Supporting this concept, exploratory analyses from pivotal clinical trials (e.g., MONALEESA and PALOMA-3) suggest that patients in the experimental arm with inactivating RB1 mutations derive less survival benefit from CDK4/6 inhibitors compared with those with RB1–wild-type tumors, while this effect was not seen in the placebo arm [154].

In efforts to identify predictive biomarkers, several candidates have been proposed. Cyclin D overexpression or amplification, particularly in the context of CDKN2A inactivation, may enhance sensitivity to CDK4/6 inhibitors. In contrast, alongside RB1 loss, increased E2F activity, and activation of cyclin E–CDK2 complexes (that resemble the triple-negative breast cancer phenotype and trigger transition from G1 to S phase independently) can circumvent CDK4/6 inhibition and drive resistance. Nevertheless, estrogen receptor (ER) positivity remains the only biomarker validated for routine clinical practice [155,156], evidence shows us that HR+ tumors express higher cyclin D1 levels and/or CDKN2A inactivation [91]. Female steroid hormones upregulate cyclin D and activate the phosphatidylinositol 3-kinase (PI3K)/AKT/mTOR signaling pathway, both of which promote tumor cell proliferation through activation of E2F transcription factors that drive DNA replication. CDK4/6 inhibitors exert their effect by disrupting the dysregulated CDK4/6–Rb–E2F axis, thereby restoring cell cycle control and inhibiting proliferation, particularly in hormone-dependent breast cancer [157,158]. High expression of cyclin E–CDK2 could deprive patients of survival benefit from CDK inhibitors; for example, while palbociclib showed worse outcomes in patients with high cyclin E1 (CCNE1) RNA expression, in the placebo group, this survival disadvantage of CCNE1 transcription was not found [159]. The clinical implementation of these biomarkers for response prediction is limited by several challenges, foremost among them the lack of methodological standardization.

One resistance mechanism is mutation in the FGFR receptor, especially for palbociclib, resulting in alternative signaling pathways, making tumors more resistant to palbociclib by enhancing downstream signaling [160]. In addition, the efficacy of CDK inhibitors depends on intact tumor suppressor pathways (e.g., Hippo and TP53 pathways), and their loss is associated with poorer prognosis [161]. For instance, functional p53 supports the efficacy of CDK4/6 inhibitors by limiting phosphorylation of the pRB, thereby halting the cell cycle. Activation of alternative oncogenic pathways, such as the PI3K–AKT–mTOR axis (frequently associated with PTEN loss), is linked to poorer prognosis and contributes to CDK4/6 inhibitor resistance [162], thereby providing a rationale for combination strategies to overcome resistance. Targeting this pathway offers a strategy to overcome resistance, as demonstrated in the phase III INAVO120 trial, which showed an overall survival benefit with inavolisib plus palbociclib in endocrine-resistant disease [163].

**Figure 5:**
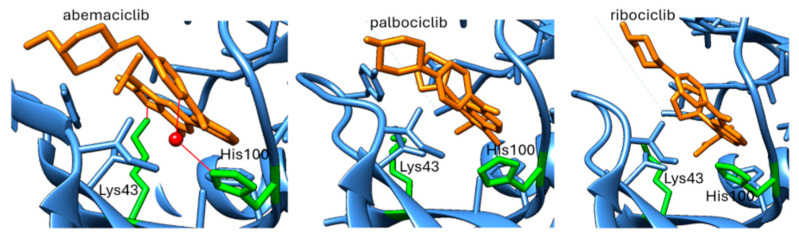
Structural comparison of abemaciclib, palbociclib, and ribociclib in the CDK6 binding site. CDK6 is shown in blue, the inhibitors in orange, and selected pocket residues in green. Although the three inhibitors occupy the same ATP-binding pocket, the figure highlights subtle differences in local pose and residue engagement. Abemaciclib is shown in association with Lys43 and a water-mediated interaction involving His100 (red sphere), whereas palbociclib and ribociclib adopt somewhat different local interaction patterns.

As already mentioned, a recognized mechanism of resistance to CDK4/6 inhibitors is the activation of cyclin E/CDK2 signaling, which can bypass dependence on CDK4/6 (Figure 1). Comparative profiling studies indicate that abemaciclib has a broader CDK inhibitory spectrum than palbociclib or ribociclib, including activity against CDK2/cyclin A/E, whereas palbociclib and ribociclib are more selective for CDK4/6 [164]. Structural studies of CDK4/6 inhibitors suggest that abemaciclib engages the ATP pocket somewhat differently from palbociclib and ribociclib [51]. In CDK6, abemaciclib forms an interaction with the catalytic Lys43 residue, buries two fluorine atoms against the ATP-binding pocket, and uses a water-mediated contact involving His100 (Figure 5). These features are not present in palbociclib or ribociclib and could point to a distinct pharmacologic profile (Figure 5). However, these observations are based on CDK4/CDK6 structures and should not be overinterpreted as direct structural proof of superior CDK2 targeting. The most notable structural variation lies in their substituents: ribociclib contains a bulky dimethylamide group, and palbociclib features an acetyl group, whereas abemaciclib is distinguished by two fluorine atoms extending into the ATP-binding pocket [88,165,166].

Another possible link comes from a biophysical study of CDK2 allostery [167]. This study indicates that cyclin binding is coupled to conformational changes in CDK2, including a shift toward a more active-like T-loop-out state (Figure S1). Within this framework, abemaciclib differed from palbociclib and ribociclib; specifically, abemaciclib promoted the T-loop-out conformational ensemble, and its interaction with CDK2 was strengthened by cyclin binding, consistent with positive cooperativity. Palbociclib and ribociclib, in contrast, did not show the same extent of conformational shift or cyclin-linked cooperativity.

Taken together, these findings provide a plausible preclinical explanation for why abemaciclib may retain activity in some tumors with CDK2/cyclin E-mediated escape, but they do not establish that abemaciclib generally overcomes resistance.

**Figure 6:**
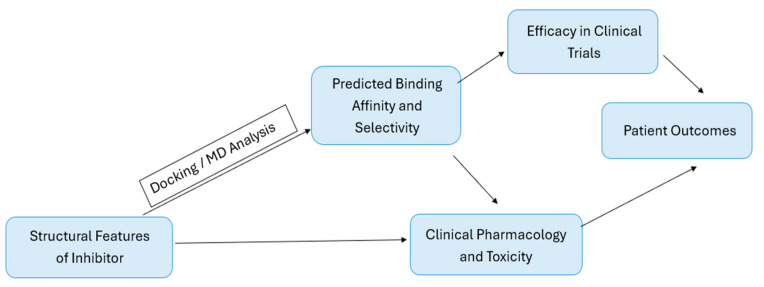
Schematic representation of the integrative framework linking structural features of CDK4/6 inhibitors with computational analyses and clinically relevant parameters. Molecular docking and molecular dynamics are shown as approaches that help relate structural properties to predicted binding affinity and selectivity, which may be considered in the broader context of efficacy, pharmacology, and toxicity.

Although these structural observations may help explain some differences in inhibitor behavior and resistance-related profiles, they provide only a limited view of the dynamic and mechanistic features that may also influence CDK4/6 inhibitor activity [168]. In this context, computational approaches have become particularly valuable, as they allow the investigation of conformational transitions, binding-pocket flexibility, and interaction patterns that are difficult to resolve from static structures alone [169]. In addition, docking-based and related in silico methods are widely used to characterize potential CDK4/6 inhibitors in terms of binding mode, predicted affinity, and selectivity [170]. The following section, therefore, discusses these computational approaches as an important complement to structural and clinical observations, and Figure 6 provides a schematic summary of this integrative framework.

## 6. Application of In Silico Methods in CDK4/6 Research

Computer-aided approaches have become indispensable in modern drug development, as they facilitate the prediction of biological activity, the detailed analysis of molecular interactions, and the rational design of new therapeutic candidates. These techniques allow for the study of complex biological systems at an atomistic level, reduce the cost and time associated with experimental validation and provide active guidance for experimental design through detailed predictions of molecular interactions [171]. Moreover, they support the assessment of the effects of mutations on ligand binding affinity, which is crucial for understanding interindividual differences in therapeutic response based on the individual genome (pharmacogenomics) and for elucidating the mechanisms underlying drug resistance [172].

The most commonly used methods in computational drug development include molecular docking, molecular dynamics (MD) simulations, quantum mechanical calculations, and structural modeling [173]. Molecular docking forms a theoretical computational method applied to investigate the binding process, interaction patterns, and affinity between a ligand and a receptor. Moreover, molecular docking results provide insights into the potential for selective binding of different inhibitors and thus facilitate better drug discovery and development [18,68]. MD simulations, on the other hand, are applied to study the dynamic interactions between receptors and ligands and provide important insights into the stability of complexes and conformational changes upon binding [174].

The described computational techniques have greatly accelerated the search for and the understanding of CDK4/6 inhibitors over the past decade. The research has increasingly focused on optimizing existing drugs (palbociclib, ribociclib, and abemaciclib) and on discovering new natural or synthetic compounds with enhanced therapeutic profiles. In this review, we summarize representative computational studies on CDK4/6 published between 2015—the year when the first selective inhibitor was approved—and 2025, with a particular focus on breast cancer research.

### 6.1. Limitations and Challenges of In Silico Approaches in CDK4/6 Research

Although in silico methods provide useful structural and mechanistic hypotheses, their results should be interpreted with appropriate methodological caution. In molecular docking, scoring functions are commonly used for pose ranking and ligand prioritization, but accurate prediction of binding affinity remains challenging. Docking scores should not be treated as direct measures of compound activity, and results obtained with different docking programs are not directly comparable because they depend on the scoring function, force field, and protocol applied [175,176,177].

For molecular dynamics simulations, important limitations include severe time-scale constraints, limited force-field accuracy, and high computational cost. Many biologically relevant processes occur on timescales beyond those accessible to conventional all-atom MD, while the reliability of simulations is fundamentally limited by force-field accuracy. These factors can lead to incomplete sampling of relevant conformational states and make direct comparison across studies difficult, particularly when different force fields, simulation lengths, or sampling protocols are used [178,179].

MM/GBSA and MM/PBSA are widely used end-point methods for estimating ligand-binding affinities, but they contain several approximations. Their performance depends on model parameters, including dielectric settings, and the methods have been applied to different systems with varying success. They are more useful for ranking relative ligand-binding affinities than for absolute binding free-energy prediction, while continuum electrostatics models also ignore the molecular structure of the solvent. In addition, the reported results may be influenced by system-dependent properties, including binding-site features, conformational relaxation upon binding, and charge distribution, which complicates direct comparison across studies [180,181,182].

High in vitro potency does not necessarily predict in vivo benefit, and generic predictions of in vivo efficacious concentration based on in vitro potency may be highly variable. Pharmacokinetic properties, target localization, active metabolites, receptor occupancy, and target turnover influence the relationship between in vitro potency and clinically efficacious concentrations [183].

These methods are primarily intended to predict binding mode and estimate binding affinity, but they do not address other determinants of in vivo efficacy, such as tissue distribution, receptor occupancy, target localization, and target turnover. Consequently, favorable predicted binding does not necessarily translate into in vivo benefit [183,184,185].

### 6.2. Molecular Dynamics Studies of CDK4/6 Inhibitors

**Figure 7:**
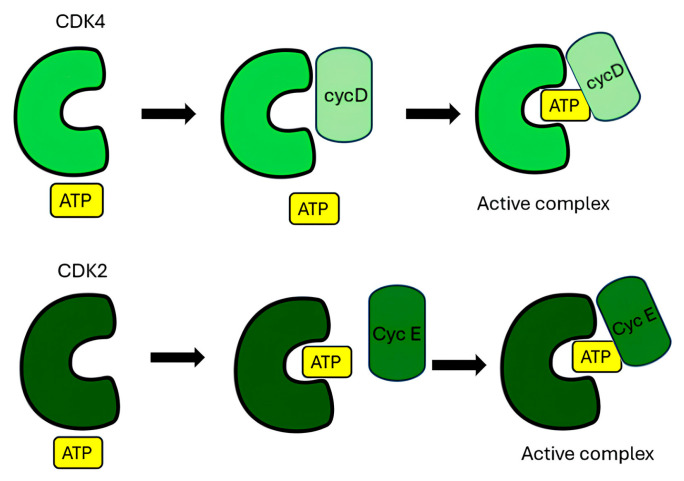
Activation mechanisms of CDK4 and CDK2. CDK4 requires prior binding of cyclin D to open the ATP binding site, whereas CDK2 can already bind ATP in its inactive state, the active conformation being stabilized by the binding of cyclin E. The figure is based on the work of Zhang et al. (2023) [186].

To elucidate the functional differences and activation mechanisms of cyclin–CDK complexes, Zhang et al. (2023) [186] performed 2 μs all-atom explicit-solvent MD simulations of cyclin-D–CDK4 and cyclin-E–CDK2 complexes using NAMD [187] (NAnoscale Molecular Dynamics) and the CHARMM36m [188] (Chemistry at HARvard Macromolecular Mechanics) force field. Their results showed that CDK2 preferentially loads ATP before binding cyclin E, while CDK4 tends to bind cyclin D first (Figure 7), resulting in a slower activation process. CDK2 exhibited a more flexible activation loop and a greater tendency for ATP loading, whereas CDK4 displayed stronger hydrophobic interactions between the T-loop (Figure 2) and the αC-helix, hindering the inward movement and reducing ATP accessibility. Activation of both kinases involves a conformational change in the T-loop, which exposes the active site of the enzyme and allows binding of ATP or CDK4/6 inhibitors. As a result, CDK4 primarily governs the progression through the G1 cell cycle phase, while CDK2 regulates the short G1/S transition with faster activation kinetics [186].

**Figure 8:**
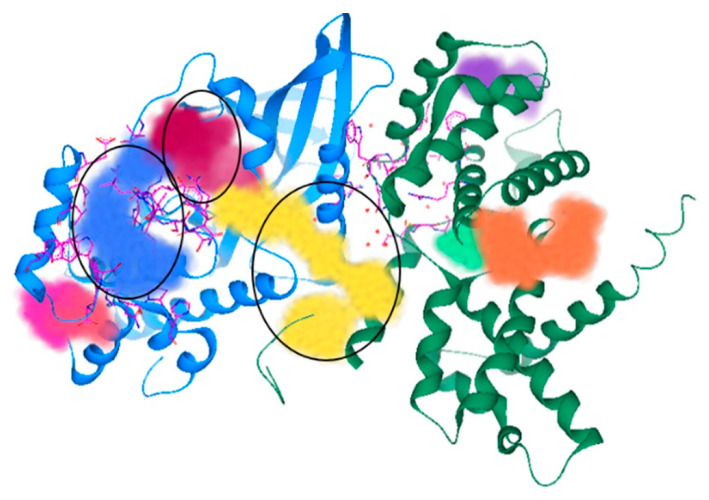
3D structures of CDK4 (blue) in complex with cyclin D (green), showing the three main binding cavities identified by Lokhande et al. (2024) [41]: two are located on the CDK4 subunit (blue and red) and one at the CDK4–cyclin D interface (yellow). The black circles highlight these identified main binding cavities, while the purple, light green, and orange regions indicate additional binding sub-pockets. The figure is based on the structural analysis presented in their study [41].

In addition to the functional aspects of activation, the structural features of the cyclin D–CDK4 complex were investigated in a further study. Lokhande et al. (2024) [41] aimed to identify potential therapeutic targets in the three-dimensional structure of the complex. Using the crystal structure of the CDK4–cyclin D complex (PDB ID: 2W96) [101] and SeeSAR [189] software (See Structure–Activity Relationships), they identified three main binding cavities within the complex (Figure 8) and nine additional sub-pockets. The first two cavities were associated with CDK4 alone, while the third was located at the CDK4–cyclin D interface. This third pocket was proposed as a potential target due to its ample number of hydrogen-bond donor and acceptor sites capable of interacting with small molecules [41].

**Figure 9:**
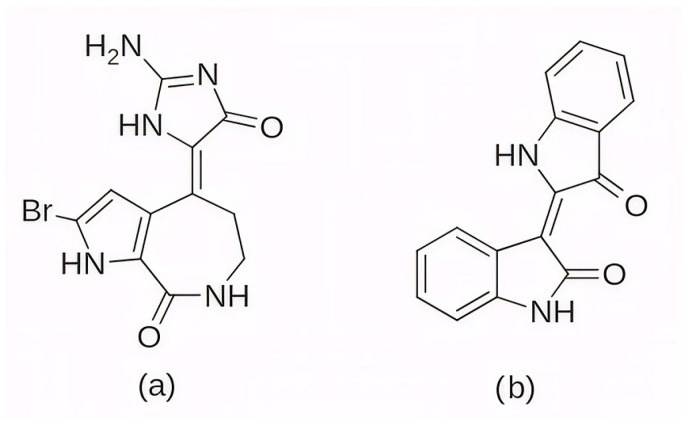
2D structures of compounds hymenialdisine (**a**) and indirubin (**b**), as reported in the study by Basati et al. (2019) [190].

Among the earlier comparisons of natural and clinically approved CDK6 inhibitors, the study by Basati et al. (2019) [190] stands out, as the authors evaluated the binding profile, structural effects, and conformational changes in CDK6 upon complexation with three inhibitors: abemaciclib, indirubin, and hymenialdisine—a marine sponge alkaloid (the structures of hymenialdisine and indirubin are shown in Figure 9, while that of abemaciclib is depicted in Figure 3). To investigate the formed interactions, MD simulations were performed using GROMACS [191] (GROningen MAchine for Chemical Simulations), and the G43A1 force field. Each simulation ran for 10 ns at 300 K. In parallel, molecular docking was carried out using AutoDock [192] with the Lamarckian Genetic Algorithm. Docking results revealed similar binding energies for indirubin (−8.33 kcal/mol) and abemaciclib (−8.30 kcal/mol), whereas hymenialdisine exhibited a weaker binding (−6.25 kcal/mol). Notably, abemaciclib showed the greatest tendency to bind to CDK6 via interaction with 16 residues in the binding site with hydrogen bonds and hydrophobic interactions. Further analyses showed that, upon complex formation, hymenialdisine and indirubin increased the total energy and reduced the radius of gyration (Rg) of CDK6. In particular, hymenialdisine significantly decreased the coil content, and other variations in the α-helical and β-sheet structures attest that these inhibitors affect the secondary structure of CDK6, leading to conformational changes that suppress its functional activity [190]. However, these results should be interpreted cautiously, as the MD simulations were limited to 10 ns, which is relatively short for CDKs and represents a flexible kinase system. This timescale may be insufficient for adequate conformational sampling and for capturing slower, functionally relevant transitions.

**Figure 10:**
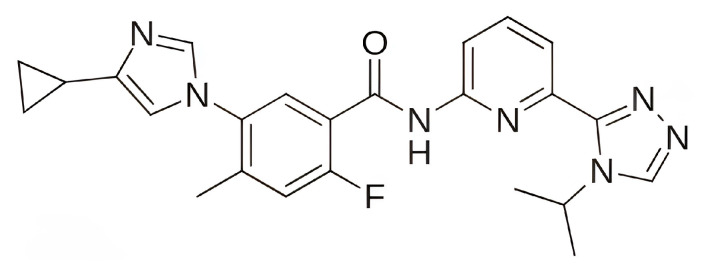
2D structure of selonsertib, as reported in the study by Baig et al. (2022) [193].

The focus on CDK6 as a therapeutic target was also emphasized in the study by Baig et al. (2022) [193], in which the inhibitory potential of selonsertib (Figure 10), a known ASK1 (apoptosis signal-regulating kinase1) inhibitor, was evaluated as a part of a drug repurposing approach. Molecular docking was performed using InstaDock [194] with the Lamarckian Genetic Algorithm and revealed that selonsertib binds to the active site of CDK6 with a binding energy of −10.9 kcal/mol. The complex was stabilized by several hydrophobic interactions, with Asp163 forming a hydrogen bond with selonsertib. To further assess the structural stability, 100 ns MD simulations were performed in GROMACS 4.6.7 using the GROMOS96 [195] (GROningen MOlecular Simulation) force field. The results showed that selonsertib binding led to a more compact and stable enzyme conformation, as evidenced by reduced RMSD values (average 2.3 Å), a lower radius of gyration (Rg), and a decreased solvent-accessible surface area (SASA). Binding affinity analysis using the MM/PBSA method yielded a binding free energy of −18.09 ± 0.36 kcal/mol. Functional assays further confirmed the inhibitory effect, showing that selonsertib indeed suppressed CDK6 kinase activity with an estimated IC_50_ of approximately 9.8 µM, calculated with an online tool provided by AAT Bioquest (Sunnyvale, CA, USA) [193,196]. However, a major limitation of this approach is that all computational and experimental analyses were conducted exclusively with selonsertib, without including any known CDK6 inhibitors (such as palbociclib, ribociclib, or abemaciclib) as reference compounds, which prevents direct benchmarking of its inhibitory potential and may limit the interpretability of the results.

**Figure 11:**
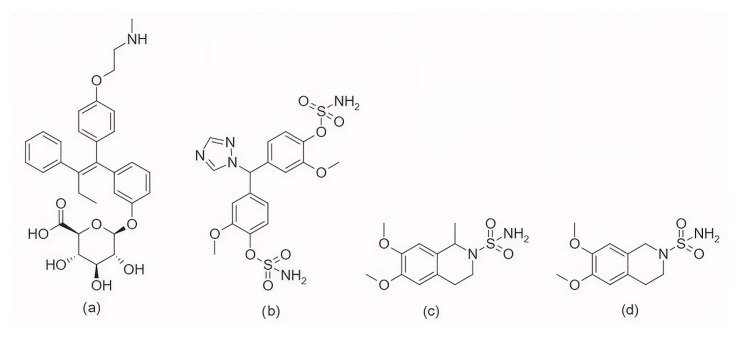
2D structures of compounds candidate 1 (**a**), candidate 2 (**b**), candidate 3 (**c**), and candidate 4 (**d**), as reported in the study by Adon et al. (2023) [197].

In the search for agents with an enhanced therapeutic potential, Adon et al. (2023) [197] investigated the simultaneous inhibition of two key regulators of the cell cycle—CDK6 and the enzyme aromatase. Using virtual screening, they developed separate structure-based pharmacophore models for each target and achieved a high predictive accuracy (ROC AUC = 0.909 for CDK6; ROC AUC = 0.815 for aromatase). The screening of the sc-PDB database [198] resulted in the identification of 755 compounds, from which four candidates (1–4; Figure 11) displayed favorable binding to both targets. Molecular docking was then carried out using the CDOCKER [199] protocol in BIOVIA Discovery Studio to evaluate the binding affinities of these compounds toward CDK6 (PDB ID: 5L2S) [106] and aromatase (PDB ID: 3S7S) [200]. Candidate 3 exhibited the lowest CDOCKER energy for CDK6 (−31.82 kcal/mol), outperforming the reference inhibitor abemaciclib (−27.35 kcal/mol). For aromatase, candidate 4 showed the most favorable binding energy (−30.35 kcal/mol), markedly better than the reference compound exemestane (−22.01 kcal/mol). The docking analysis revealed that the newly identified candidates form key interactions comparable to those of the reference drugs. The stability of the docked complexes was further evaluated through 50 ns MD simulations using the Desmond [201] module of the Schrödinger suite. Each complex was solvated in an explicit TIP3P water box with a 10 Å buffer region, neutralized with counterions, and subjected to NPT ensemble conditions (300 K, 1 bar) applying the OPLS3e (Optimized Potentials for Liquid Simulations) force field. Candidate 1 showed average RMSD values of about 2.2 Å for CDK6 and 3.0 Å for aromatase. Candidate 2 displayed similarly stable trajectories (≤2.1 Å for CDK6 and ≤1.2 Å for aromatase), while candidates 3 and 4 also maintained equilibrium throughout the simulations, with RMSD values generally below 3 Å for CDK6 and below 6 Å for aromatase. These results support the conformational stability of the docked complexes and suggest that candidates 1, 2, 3, and 4 could be possible dual inhibitors of CDK4/6 and aromatase. However, these findings are based on computational chemistry techniques and therefore require further validation through biochemical, in vitro, and in vivo studies. Nevertheless, the potential therapeutic benefit of dual CDK6–aromatase inhibition should be interpreted cautiously in view of toxicity, since the in silico ADMET analysis in the original study indicated that all identified candidates have the potential to be hepatotoxic [197]. Although multitarget drugs may offer advantages such as a reduced risk of drug–drug interactions, broader efficacy, enhanced patient convenience, and decreased treatment complexity, their potential benefits must be weighed against important limitations, including drug promiscuity and off-target interactions, which may increase the risk of toxicity, adverse effects, unintended pharmacological responses, and ultimately clinical failure [202].

**Figure 12:**
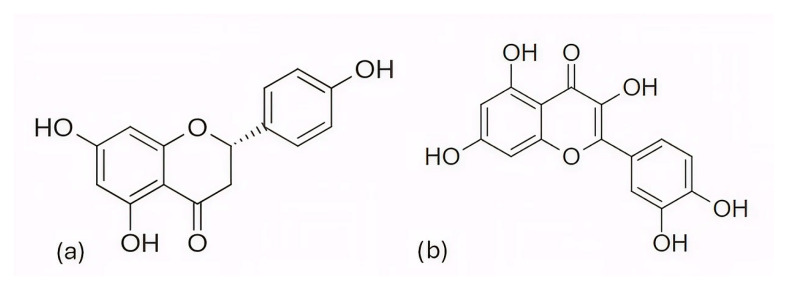
2D structures of compounds naringenin (**a**), as reported in the study by Yousuf et al. (2022) [203], and quercetin (**b**), as reported in the study by Yousuf et al. (2020) [207].

Among the natural compounds identified as potential CDK6 inhibitors, the inhibitory potential of naringenin (NAG; Figure 12a) was investigated in the study by Yousuf et al. (2022) [203], which reported a binding energy of −7.51 kcal/mol for the CDK6 complex based on molecular docking. The docking was performed using the Glide XP [204] module from the Schrödinger suite, with a receptor grid centered on the CDK6 active site (PDB ID: 3NUP) [108]. To validate the binding mode, 200 ns MD simulations were conducted using AMBER18 [205] (Assisted Model Building with Energy Refinement) with the FF14SB force field [206]. The NAG–CDK6 complex showed increased structural stability compared to apo CDK6, as indicated by a lower average RMSD (1.89 Å vs. 2.09 Å), slightly lower Rg (20.11 Å vs. 20.19 Å), and SASA (14,251 Å^2^ vs. 14,289 Å^2^). MM/GBSA calculations further pointed to a strong inhibition, with a binding free energy of −45.36 kcal/mol. Fluorescence analysis yielded a binding constant of 3.55 × 10^6^ M^−1^, while isothermal titration calorimetry produced a Ka value of 7.06 × 10^6^ M^−1^. Functional validation using an ATPase assay also confirmed that NAG indeed inhibits CDK6 with an IC_50_ of 3.13 µM. Finally, cell-based functional studies demonstrated that NAG decreased the viability of human cancer cell lines (A549 and MCF-7), induced apoptosis, and reduced their colony formation ability [203]. A similar effect was confirmed for quercetin (Figure 12b), a flavonoid compound identified among a set of natural molecules (gallic acid, ferulic acid, caffeic acid, rosmarinic acid, capsaicin, tocopherol, limonene, and quercetin) as the most potent CDK6 binder in a screening study by Yousuf et al. (2020) [207]. In the initial molecular docking screen, quercetin exhibited the lowest binding free energy (−8.6 kcal/mol) and the highest binding constant (1.3 × 10^7^ M^−1^), indicating a markedly stronger interaction compared with the other tested natural compounds (ΔG ranging from −6.3 to −7.7 kcal/mol, K values below 10^4^ M^−1^). A refined docking analysis using the validated CDK6 structure (PDB ID: 3NUP) further confirmed stable binding with an energy of −5.87 kcal/mol and the formation of hydrogen bonds with residues Asp102 and Asp163. The 200 ns MD simulations generated in GROMACS with the GROMOS96 53a6 force field [208] showed conformational changes within the active site due to binding. In vitro assays confirmed an IC_50_ value of 5.89 µM, along with decreased CDK6 expression, increased apoptosis in MCF-7 and A549 cells, and reduced ROS production [207].

**Figure 13:**
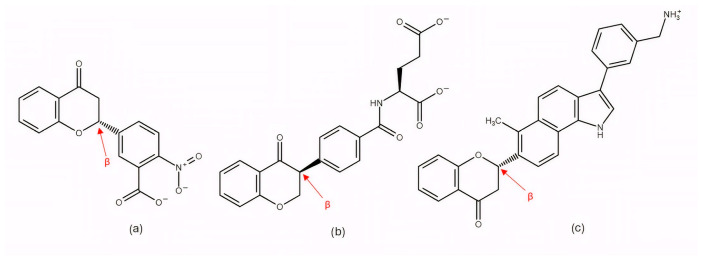
2D structures of the optimized flavanone lead compounds 20 (**a**), 25 (**b**) and 29 (**c**). The red arrows indicate the β-position at which structural modifications were introduced, as reported by Nagare et al. (2023) [209].

Further research into improving the binding affinity of flavonoids has focused on the structural optimization of flavanones, a class of natural substances found primarily in citrus fruits. In the study by Nagare et al. (2023) [209], flavanones were chemically modified at the β-position (Figure 13) and analyzed in complex with the cyclin D/CDK4 receptor. The protein structure of the cyclin D/CDK4 complex was obtained from the Protein Data Bank (PDB ID: 2W9Z) [101] and its binding site was identified using FlexX version 2.3.2 [210] docking. MD simulations of 100 ns performed with Desmond software showed flavanone derivatives 20, 25 and 29 (Figure 13) forming stable complexes with the receptor, as shown by trajectory analysis. The simulations revealed that flavanone 20 maintained the highest structural stability (RMSD ≈ 5 Å) and formed hydrogen bonds with residues Lys35, Val96, Lys142, and Asn145, hydrophobic interactions with Ile12, Val20, Ala33, Val72, Asn145, and Leu147, as well as ionic interactions with Lys142 and water bridges with Gly13, Val14, Gly15, and Asp158 (Figure 2), indicating a thermodynamically stable complex. Among the tested compounds, flavanone 20 exhibited the most favorable binding free energy (−51.607 ± 3.681 kcal/mol), followed by flavanone 25 (−45.236 ± 3.970 kcal/mol) and flavanone 29 (−42.656 ± 3.296 kcal/mol), showing stronger binding affinities than the reference drug palbociclib (−25.129 kcal/mol) and thereby supporting their potential as selective CDK4 inhibitors [209].

**Figure 14:**
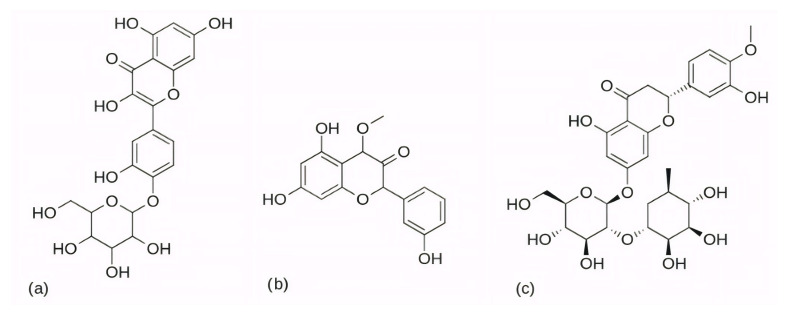
2D structures of compounds quercetin-4′-glucoside (**a**), 3,5,7-trihydroxy-4′-methoxyflavone (**b**), and hesperetin-7-O-neohesperidoside (**c**), as reported in the study by Abo-Elghiet et al. (2022) [211].

Further confirmation of the inhibitory potential of flavonoids was provided by Abo-Elghiet et al. (2022) [211], who identified 28 flavonoid constituents in the chloroform/methanol (90/10) extract of *Viscum cruciatum* using LC-MS/MS analysis. Among them, three compounds—quercetin-4′-glucoside, 3,5,7-trihydroxy-4′-methoxyflavone, and hesperetin-7-O-neohesperidoside (Figure 14) were selected for further computational and experimental evaluation. Molecular docking was performed using AutoDock 4.2 to predict their binding modes for CDK2, CDK4, and CDK6. Subsequent 100 ns MD simulations, carried out in GROMACS using the GROMOS96 43a1 force field, confirmed the formation of stable complexes with all three targets. Stability was supported by low average RMSD values: 1.55 Å for CDK2–quercetin-4′-glucoside; 1.79 Å for CDK4–3,5,7-trihydroxy-4′-methoxyflavone; and 1.88 Å for CDK6–hesperetin-7-O-neohesperidoside. MM/PBSA binding free energy analysis revealed the strongest binding affinity for quercetin-4′-glucoside (−31.4 ± 1.2 kcal/mol), followed by 3,5,7-trihydroxy-4′-methoxyflavone (−26.3 ± 1.0 kcal/mol), and hesperetin-7-O-neohesperidoside (−23.6 ± 0.8 kcal/mol). The in silico results were experimentally confirmed by Western blot analysis, which showed a significant decrease in the level of CDK2, CDK4, and CDK6 protein expression in MCF-7 cells treated with the *V. cruciatum* ChMe extract at its IC_50_ concentration (23.8 μg/mL) compared to untreated cells [211].

**Figure 15:**
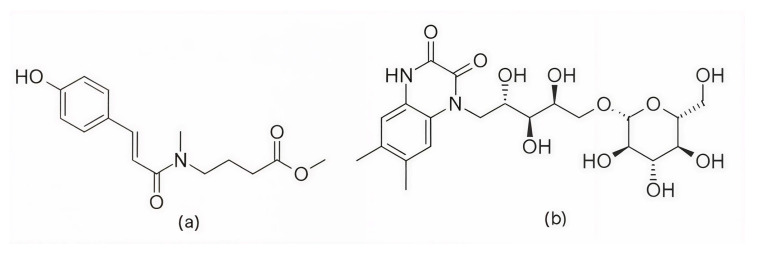
2D structures of compounds baimantuoluoamide A (**a**) and baimantuoluoamide B (**b**), as reported in the study by Gurushankar et al. (2021) [212].

Among the natural alkaloids, baimantuoluoamide A and B (Figure 15) were identified as potential CDK4 inhibitors in the study by Gurushankar et al. (2021) [212]. Molecular docking was performed using AutoDock Vina [213] and two CDK4 crystal structures, PDB ID: 2W9Z [101], which was selected for docking due to its structural completeness, and PDB ID: 1GII [214], which provided the conformation of the reference inhibitor 1PU. The resulting binding poses served as input for 300 ns MD simulations performed in AMBER16 at 310 K and 1 atm. Quantitative analysis using the MM/GBSA method revealed that baimantuoluoamide B exhibited the strongest binding free energy (−50.66 kcal/mol), while baimantuoluoamide A (−24.98 kcal/mol) and the reference inhibitor 1PU (−22.47 kcal/mol) showed similar affinities. In addition, baimantuoluoamide B formed the highest average number of hydrogen bonds (4.97 ± 1.56) compared to baimantuoluoamide A (1.81 ± 0.96) and the reference 1PU inhibitor (0.58 ± 0.90), further supporting its advantage over the other compounds analyzed [212].

**Figure 16:**
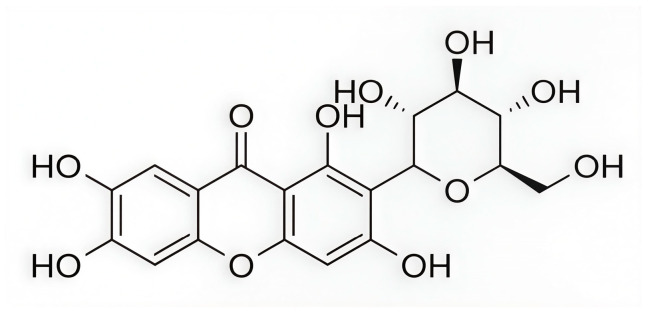
2D structure of mangiferin, as reported in the study by Ashraf et al. (2022) [215].

In a subsequent study, Ashraf et al. (2022) [215] investigated the xanthonoid mangiferin (Figure 16), which is a phytochemical compound derived from the bark and leaves of *Mangifera indica*, as a potential inhibitor of the CDK4 enzyme. It is worth noting that in their research, each selected phytochemical ligand was tested on a different target protein (luteolin on CDK2, apigenin on TP53, quercetin on HIBCH, etc.). Therefore, the results for mangiferin were limited exclusively to its complex with the CDK4 enzyme, without comparison to other potential ligands or known inhibitors of this enzyme. Molecular docking performed using AutoDock Vina through the PyRx platform [216] yielded a binding energy of −7.8 kcal/mol for the CDK4–mangiferin complex. The resulting binding pose served as the starting structure for MD simulations carried out in AMBER16 under NPT ensemble conditions at 310 K and 1 bar, employing the FF14SB force field for the protein and GAFF for the ligand. Docking results indicated that mangiferin forms non-bonded interactions with active site residues Ile12, Gly13, Val14, Gly15, Val20, Ala33, Lys35, His92, Val93, Asp94, Gln95, Asp96, Lys139, Glu141, and Leu144 (Figure 2). The binding remained stable throughout 170 ns of simulation, with the system exhibiting a low average RMSD value (2.2 ± 0.40 Å) and Rg (20.3 ± 0.20 Å). The binding free energy, calculated using the MM/GBSA method over the final 20 ns of simulation, was −20.2 ± 3.3 kcal/mol, indicating a thermodynamically favorable interaction and confirming the potential of mangiferin as a CDK4 inhibitor [215].

**Figure 17:**
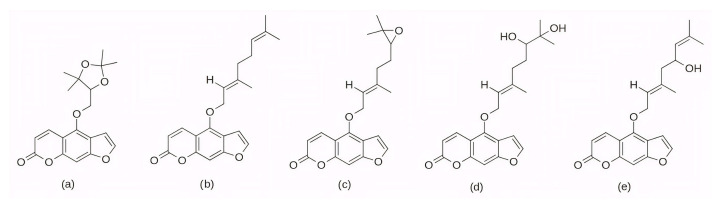
2D structures of compounds oxypeucedanin hydrate acetonide (**a**), bergamottin (**b**), epoxybergamottin (**c**), dihydroxybergamottin (**d**), and notopterol (**e**), as reported in the study by Sarma et al. (2025) [217].

A group of potential CDK4 inhibitors was further expanded by Sarma et al. (2025) [217], who employed AutoDock 4.2 with the Lamarckian Genetic Algorithm to dock 50 furanocoumarin compounds into the ATP-binding site of CDK4. Based on more negative binding energies than the control compound abemaciclib (−7.93 kcal/mol), five top-scoring ligands were selected: oxypeucedanine hydrate acetonide (−8.52 kcal/mol), bergamottin (−8.51 kcal/mol), epoxybergamottin (−8.26 kcal/mol), dihydroxybergamottin (−8.17 kcal/mol), and notopterol (−8.33 kcal/mol). The 2D structures of these five selected ligands are presented in Figure 17. For all selected complexes, 100 ns MD simulations were performed using the GROMACS software and the CHARMM36 force field. RMSD analysis of the protein backbone yielded values between 3.5 and 4.0 Å in all five protein–ligand systems, with the notopterol complex exhibiting the smallest deviation. RMSF analysis revealed reduced fluctuations in most areas of the activation segment in the complexes with epoxybergamottin, dihydroxybergamottin, and notopterol, while bergamottin and oxypeucedanine hydrate acetonide showed increased mobility in the same region. Subsequent MM-PBSA calculations confirmed that epoxybergamottin, dihydroxybergamottin, and particularly notopterol displayed the most favorable binding free energies. Finally, ONIOM [218] (so-called Our own N-layered Integrated molecular Orbital and Molecular mechanics) calculations on the notopterol–CDK4 complex demonstrated the retention of key hydrogen bonds and a significantly negative interaction energy, indicating notopterol as the most promising potential CDK4 inhibitor among the studied compounds [217].

**Figure 18:**
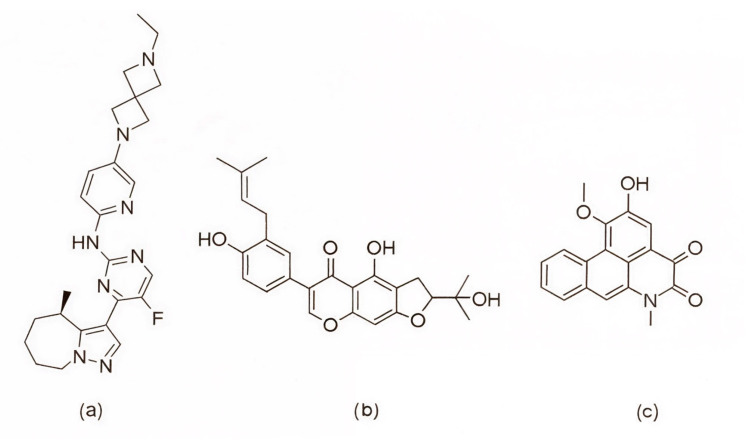
2D structures of compounds and N1J (**a**), IMPHY002642 (**b**), and IMPHY005260 (**c**), as reported in the study by Khatoon et al. (2025) [219].

In a study focusing on CDK6, Khatoon et al. (2025) [219] screened approximately 12,000 phytochemical compounds from the IMPPAT 2.0 database [220] using molecular docking with AutoDock Vina. To validate the docking protocol, the CDK6 protein was first re-docked with its co-crystallized reference ligand N1J (Figure 18a), which showed a binding energy of −9.2 kcal/mol. Two ligands, IMPHY002642 (Figure 18b) and IMPHY005260 (Figure 18c), were then selected for further analysis based on their stronger binding affinities of −10.6 and −10.5 kcal/mol, respectively. The higher affinity values of IMPHY002642 and IMPHY005260 indicated a more stable interaction with the CDK6 binding site compared to the reference ligand. Both compounds were subsequently subjected to 300 ns MD simulations using GROMACS and the CHARMM36 force field. RMSD analyses revealed stable complexes with values of 1.98 Å for IMPHY002642 and 1.91 Å for IMPHY005260, comparable to the apo form of the protein (1.91 Å). RMSF values ranged from 0.11 to 0.13 nm, showing minimal residue-level fluctuations upon ligand binding. The average radius of gyration (Rg) remained consistent at 2.01 nm for the unbound protein and 2.03 nm and 2.02 nm for the complexes with IMPHY002642 and IMPHY005260, respectively, suggesting no major conformational expansion. Similarly, SASA values decreased slightly from 154.23 nm^2^ (apo CDK6) to 153.22 nm^2^ and 152.67 nm^2^ after ligand binding, indicating increased compactness and stability of the complexes. IMPHY002642 formed stable hydrogen bonds with Val91, Lys137 (two), and Val170, while IMPHY005260 interacted with Glu89, His90, Val91, Gln93, Asp94, and Gln139 (Figure 2). The selection of these two ligands was further supported by favorable ADMET and PASS results, indicating good absorption, low predicted toxicity, and bioactivities consistent with anticancer potential, including antioxidant and anti-inflammatory effects. Taken together, their strong binding affinities, stable molecular interactions, and favorable pharmacological properties highlight IMPHY002642 and IMPHY005260 as promising natural candidates for CDK6 inhibition [219].

**Figure 19:**
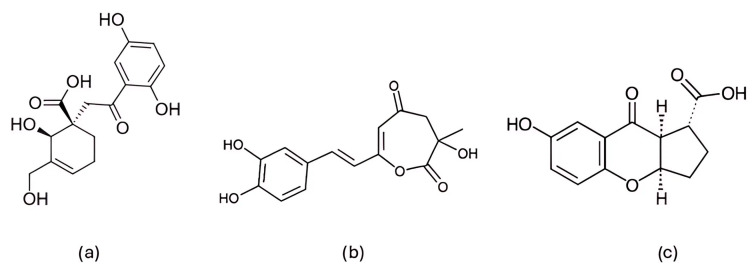
2D structure of the lead compounds MSID000025 (**a**), MSID001071 (**b**), and MSID000040 (**c**), as reported in the study by Debnath et al. (2024) [221].

Debnath et al. (2024) [221] performed a virtual screening of potential natural CDK4/6 inhibitors using the MeFSAT database [222], which contains secondary metabolites from fungi. From an initial set of 411 compounds, 191 met Lipinski’s rule of five, and 28 exhibited favorable pharmacokinetic properties based on SwissADME [223] predictions, and 21 were further selected according to OSIRIS DataWarrior [224] non-toxicity predictions. Consensus molecular docking was carried out using seven different programs—AutoDockTools, idock [225], LeDock [226], Qvina2 [227], Smina [228], AutoDock Vina, and rDock [229], and yielded six top candidates, with compound MSID000025 (Figure 19a) emerging as the most promising. The stability of the complexes was indicated by 200 ns MD simulations performed in GROMACS using the CHARMM36 force field, with ligand force field parameters generated via the CGenFF [230] webserver, TIP3P water model, and standard NVT/NPT equilibration. MD confirmed complex stability relative to the apo-CDK4/6 reference (RMSD = 3.05 ± 0.37 Å, Rg = 20.26 ± 0.17 Å), with MSID000025 maintaining a similarly stable conformation (RMSD = 3.47 ± 0.23 Å, Rg = 20.10 ± 0.15 Å) and a lower solvent-accessible surface area (153.75 ± 3.90 nm^2^ vs. 159.38 ± 3.23 nm^2^ for the reference). MSID000025 formed persistent hydrogen bonds with Glu144 and Leu166 and hydrophobic contacts with Ala132 and Ile149, whereas MSID001071 (Figure 19b) and MSID000040 (Figure 19c) showed slightly higher flexibility but retained stable binding throughout the simulation. Functional evaluation using the MTT assay on MCF-7 cells confirmed cytotoxic activity, with MSID000025 exhibiting the lowest IC_50_ value (13.55 ± 0.62 µM) compared to MSID001071 (26.24 ± 1.48 µM), supporting its potential as a lead natural CDK4/6 inhibitor candidate [221].

**Figure 20:**
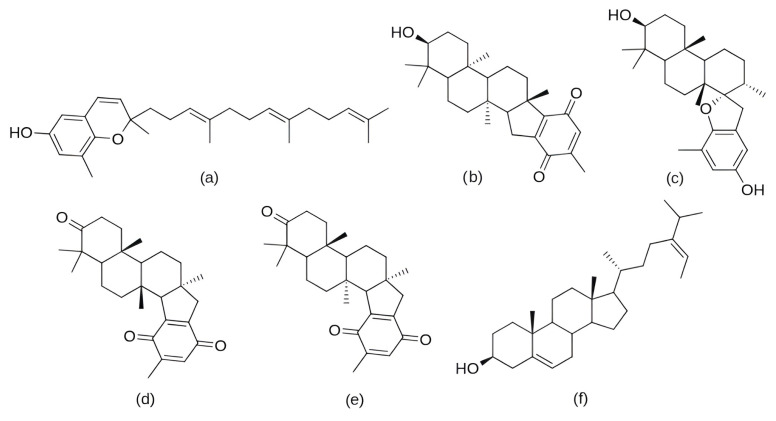
2D structures of compounds sargaol (**a**), flabellinone (**b**), stypodiol (**c**), atomarianone A (**d**), atomarianone B (**e**), and fucosterol (**f**), as reported in the study by Demirkıran et al. (2024) [69].

In addition to fungi, marine organisms have also been recognized as a valuable source of natural compounds with diverse biological activities. In the study by Demirkıran et al. (2024) [69], six known compounds (Figure 20a–f)—five meroterpenoids (sargaol, flabellinone, stypodiol, atomarianone A, and atomarianone B) and one steroid (fucosterol)—were isolated from the brown alga *Stypopodium schimperi*, collected from the Kaleiçi coast of Antalya, Türkiye. Their cytotoxicity was evaluated using the MTT assay on two breast cancer cell lines (MCF-7 and MDA-MB-231) and one human fibroblast cell line (CCD-1079Sk). Among the tested compounds, fucosterol exhibited the highest selectivity, with IC_50_ values of 12.30 ± 0.38 µM (MDA-MB-231) and 15.50 ± 1.11 µM (MCF-7), and no significant cytotoxicity on healthy fibroblasts (IC_50_ > 200 µM). Subsequently, molecular docking was performed using Schrödinger’s Induced Fit Docking (IFD) protocol with Glide XP against eight breast cancer-related targets, including ERα, HER2, EGFR, VEGFR1, VEGFR2, and the cyclin-dependent kinases CDK2, CDK4, and CDK6. In the following, only the results for CDK4 and CDK6 are discussed. Fucosterol showed favorable binding affinities toward both kinases, with docking scores of −10.16 kcal/mol for CDK4 and −10.37 kcal/mol for CDK6. For comparison, the reference CDK4/6 inhibitor palbociclib exhibited docking scores of −10.35 kcal/mol (CDK4) and −9.69 kcal/mol (CDK6), indicating that fucosterol binds to both kinases with comparable or slightly better predicted affinity. MD simulations of 100 ns, performed using the Desmond software and the OPLS3e force field, further indicate the stability of these complexes. The binding free energies calculated by the MM-GBSA method were −62.58 kcal/mol for the CDK4–fucosterol complex and −77.00 kcal/mol for the CDK6–fucosterol complex, compared to −80.90 kcal/mol and −88.22 kcal/mol obtained for palbociclib with CDK4 and CDK6, respectively. Although the interaction energies of fucosterol are slightly less favorable than those of palbociclib, they still indicate stable and energetically advantageous complex formation, highlighting fucosterol as a promising natural scaffold for CDK4/6 inhibition [69].

**Figure 21:**
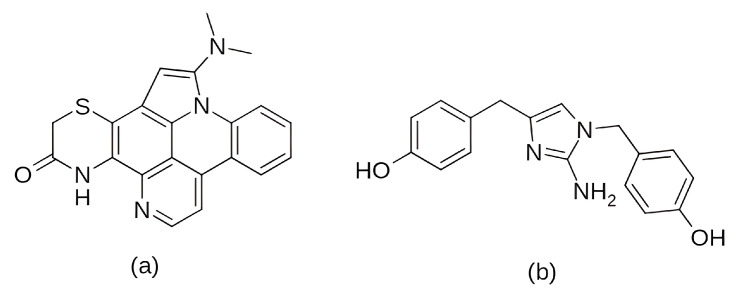
2D structures of compounds CMNPD2744 (**a**) and CMNPD11585 (**b**), identified as lead candidates (isonaamine A and cycloshermilamine D, respectively) in the study by Debnath et al. (2025) [231].

Marine sources were further explored by Debnath et al. (2025) [231], who performed a structure-based virtual screening of compounds from the CMNPD (Comprehensive Marine Natural Products Database [232]) and MNP (Marine Natural Product) library [233] to identify new potential CDK4/6 inhibitors. After a multi-stage filtering based on drug-likeness criteria, PAINS alerts, ADME properties, and toxicity profiles, 25 compounds were selected and analyzed by consensus molecular docking using algorithms AutoDockTools 4.2, idock, LeDock, Qvina 2, Smina, AutoDock Vina 1.2.0, PLANTS [234] and rDock. Based on the results obtained, six top-scoring compounds were subjected to 500 ns MD simulations in GROMACS using the CHARMM36 force field for proteins, CGenFF parameters for ligands, and the TIP3P water model. The highest structural stability was observed for the CMNPD2744–CDK6 complex (isonaamine A; Figure 21a), with an average RMSD of 2.806 ± 0.205 Å and Rg of 20.394 ± 0.134 Å. The CMNPD11585–CDK4 complex (cycloshermilamine D; Figure 21b) also demonstrated favorable stability, with an RMSD of 3.658 ± 0.359 Å and Rg of 20.423 ± 0.205 Å. Both complexes showed low RMSF values and compact structures with well-maintained solvent exposure. Throughout the simulations, both ligands maintained persistent hydrogen bonds and hydrophobic contacts with key catalytic residues such as Ile9, Val91, Leu142, Ala152, and Asp153 (Figure 2), contributing to their high structural stability and strong binding within the CDK4/6 active site. In cytotoxicity assays on MCF-7 cells, CMNPD11585 demonstrated the highest potency with an IC_50_ value of 0.03 ± 0.002 µM, exceeding that of the reference inhibitor ribociclib (0.061 ± 0.006 µM), while CMNPD2744 showed a similar IC_50_ value of 0.073 ± 0.003 µM. These findings suggest that both natural compounds exhibit inhibitory potential on par with, or exceeding, the clinically approved CDK4/6 inhibitor, highlighting their promise as lead structures for further drug development [231].

Among the marine-derived compounds, bioactive peptides have also been investigated as potential CDK4 inhibitors. In the study by Wargasetia et al. (2021) [235], peptides isolated from the sea cucumber *Cucumaria frondosa* were analyzed, of which WPPNYQW and YDWRF exhibited a high affinity for the CDK4 binding site (−9.4 and −9.3 kcal/mol, respectively), surpassing that of the reference inhibitor abemaciclib (−9.0 kcal/mol). These binding affinities were obtained using molecular docking performed with AutoDock Vina integrated with PyRx. Abemaciclib formed hydrogen bonds with Gly15, Ile12, Asp99, Asp97, and Thr102, and hydrophobic interactions with Val20, Leu147, His95, Ala10, and Ile12. The WPPNYQW peptide formed seven hydrogen bonds with residues Ile12, Val14, Ala16, Tyr17, Thr177, Glu144, and Asp99, and seven hydrophobic interactions involving Leu147, Trp179, Val176, Cys215, Ile12, Ala33, and Ala157. The YDWRF peptide formed six hydrogen bonds with Asp105, Val14, Thr102, Val96, Thr177, and Asp99, and four hydrophobic interactions with Ile12, Leu147, Val20, and Ala33 (Figure 2). The stability of the complexes was further evaluated by 20 ns MD simulations in YASARA [236] (Yet Another Scientific Artificial Reality Application) under physiological conditions (37 °C, 1 atm, pH 7.4, and 0.9% NaCl). RMSD analysis showed that the WPPNYQW–CDK4 complex remained largely stable, and only transient instability was observed between 6 and 8 ns, whereas the YDWRF–CDK4 complex exhibited a greater conformational variability. In contrast, the CDK4–abemaciclib complex showed continuous RMSD fluctuations exceeding 3 Å after 4 ns, indicating that the reference inhibitor formed a less stable interaction with CDK4 than the WPPNYQW peptide [235].

**Figure 22:**
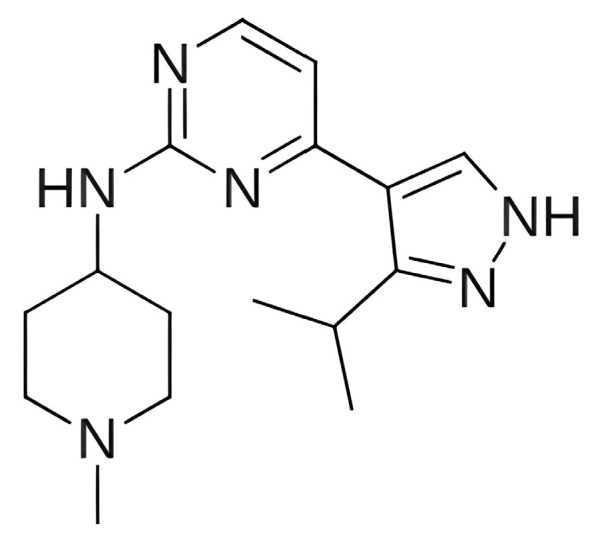
2D structure of the inhibitor A1, as reported in the study by Dong et al. (2017) [237].

In recent years, intensive research efforts have been made to develop synthetic compounds that allow for precise structural optimization to increase the selectivity of inhibitors toward their intended targets and improve pharmacological profiles. A representative example of such an approach is the study by Dong et al. (2017) [237], which provided a detailed insight into the inhibitory selectivity of 4-(pyrazol-4-yl)-pyrimidine derivatives for CDK4 over the highly homologous CDK2, which shares approximately 45% sequence identity with CDK4. Among the investigated derivatives, compound A1 (Figure 22) was analyzed using molecular docking (performed with AutoDock using the Lamarckian Genetic Algorithm), 30 ns MD simulations, and MM-PBSA free energy calculations performed in AMBER10 with ff03 and GAFF force fields. The CDK2/A1 complex exhibited a lower average RMSD value (1.80 Å) than the CDK4/A1 complex (2.60 Å), yet MM-PBSA analysis yielded a more favorable binding free energy for CDK4 (−37.76 kcal/mol vs. −30.91 kcal/mol), mainly due to stronger electrostatic interactions (−20.67 kcal/mol for CDK4 vs. −11.94 kcal/mol for CDK2). This selectivity was primarily attributed to a hydrogen bond between the NH group of the pyrazole ring and the residue Asp158. Moreover, CDK4 exhibited a greater conformational flexibility, with a 49.7° rotation in a mobile domain compared to 12.5° in CDK2 [237].

**Figure 23:**
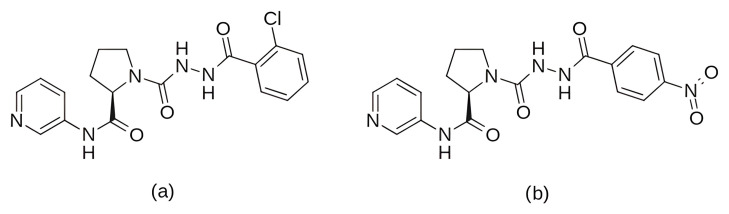
2D structures of compounds **7c** (**a**) and **7p** (**b**), as reported in the study by Liang et al. (2022) [238].

A further development of synthetic CDK4/6 inhibitors was presented by Liang et al. (2022) [238], who designed a new series of inhibitors based on the N-(pyridin-3-yl)proline scaffold, derived from the fragment N′-acetylpyrrolidine-1-carbohydrazide. In total, nineteen derivatives (**7a**–**7s**) and two enantiomers (**8c** and **8p**) were synthesized and evaluated for CDK4/6 inhibition. Among the resulting derivatives, compounds **7c** and **7p** (Figure 23) proved particularly promising. In enzymatic assays, **7c** and **7p** displayed potent inhibition of CDK4 (IC_50_ = 1.58 nM and 5.01 nM) and CDK6 (IC_50_ = 4.09 nM and 3.97 nM). Compound **7c** achieved 57.68% tumor growth inhibition in an in vivo mouse model using MCF-7 xenografts, with no observed toxic effects, while its IC_50_ value in cellular assays was 0.92 μM (compared to 2.8 μM for **7p**). The stability of their CDK4 complexes was evaluated by 20 ns MD simulations in GROMACS using the CHARMM36 force field. The results showed that the **7c** complex rapidly converged to a stable conformation with RMSD fluctuations between 0.9 and 1.2 nm, whereas the **7p** complex exhibited prolonged RMSD oscillations for about 10 ns, indicating lower structural stability. Docking analysis indicated that the inhibitor interacts with key residues of the CDK6 binding pocket, including Val101 and Lys43 through hydrogen bonds and His100 via a salt bridge, while hydrophobic residues such as Phe98, Leu152 and Ala162 contribute to pocket stabilization (Figure 2). Together, these findings demonstrate that the N-(pyridin-3-yl)proline scaffold provides a promising framework for the development of selective CDK4/6 inhibitors, with compound **7c** representing a potent, stable and well-tolerated lead candidate exhibiting strong antitumor activity both in vitro and in vivo [238].

**Figure 24:**
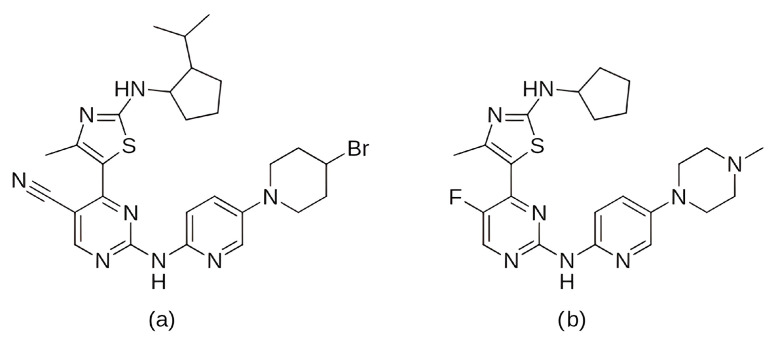
2D structures of compounds D10 (**a**) and 16 (**b**), as reported in the study by Fu et al. (2022) [239].

The development of multitarget inhibitors was also investigated by Fu et al. (2022) [239], who identified derivatives of 4-thiazol-N-(pyridin-2-yl)pyrimidin-2-amine as effective inhibitors of both CDK4 and CDK6. Molecular docking of the template compound and newly designed derivatives was performed against both kinases using the Surflex-Dock [240] module in Sybyl-X2.0 to examine their binding modes. Compounds 16 and D10 (Figure 24) emerged as the most promising candidates based on the docking scores and predicted activity. To evaluate binding stability, these compounds were subjected to 100 ns MD simulations with AMBER16 using the ff99SB force field for proteins and GAFF for ligands. After about 70 ns, the RMSD values for the CDK4/16 and CDK4/D10 complexes stabilized at ~4.25 Å and ~3.75 Å, respectively, while the CDK6 complexes exhibited a similar stability (~4.0 Å for 16 and ~3.5 Å for D10). All structures reached a metastable state in the final phase of the simulation. Interaction analysis revealed that both ligands formed stable hydrogen bonds with the key residues Val96 and Lys22 in CDK4, as well as with Val101 in CDK6 (Figure 2). Before MD simulation, an unstable hydrogen bond between compound 16 and Lys35 in CDK4 was observed, which transformed into a π–NH interaction after the simulation. Moreover, compound D10 established further stable hydrogen bonds with His100 (in both CDK4 and CDK6) and Lys43 (in CDK6), thereby enhancing the overall binding interactions between the proteins and the ligands [239].

**Figure 25:**
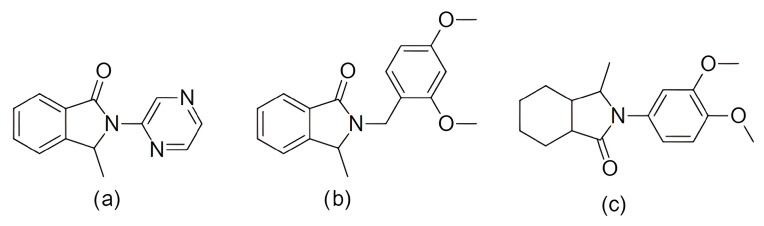
2D structures of compounds M18 (**a**), M24 (**b**), and M32 (**c**), as reported in the study by Singh et al. (2023) [241].

In the search for additional substances with a potential inhibitory effect against CDK4, compounds from the class of 3-methyleneisoindolin-1-one were investigated. Singh et al. (2023) [241] selected three of the most promising candidates—M18, M24 and M32 (Figure 25)—from an initial group of thirty-two compounds based on molecular docking results, obtained using the CDOCKER protocol in BIOVIA Discovery Studio, that exhibited stronger interaction energies compared to the others. Docking analysis revealed that all compounds formed multiple hydrogen bonds, hydrophobic, and electrostatic interactions within the CDK4 active site. Palbociclib bound mainly through Lys22, Val69, Glu91, and Ala154, whereas M18, M24, and M32 interacted with key residues such as Glu91, His92, Val93, Asp94, Asp96, Leu144, and Ala154 (Figure 2). Among them, M24 established the most extensive interaction network, suggesting a particularly strong and specific binding mode comparable to or exceeding that of palbociclib. To evaluate their binding stability, 250 ns MD simulations were performed using GROMACS v4.6.7 with the GROMOS96 43a1 force field, applying the SPC water model and system neutralization with sodium ions. The RMSD values showed that all three compounds maintained a stable conformation, with an average RMSD of about 5.2 Å; however, M24 showed a transient instability that only subsided after 128 ns, after which its trajectory stabilized and equilibrated similarly to the others. RMSF analysis confirmed low flexibility in the binding regions (0.6–2.1 Å), indicating stable ligand–receptor interactions. The MM-PBSA analysis revealed energetically favorable binding for compounds M24 (−26.40 kcal/mol) and M32 (−21.48 kcal/mol), whereas M18 (7.68 kcal/mol) and palbociclib (0.93 kcal/mol) exhibited significantly less favorable binding energies. These results indicate that the polar solvation energy contributed to the weaker affinity of palbociclib and M18, while M24’s strong van der Waals and electrostatic contributions dominated its binding strength. Additional steered MD simulations showed a greater structural stability for M24, which required the highest external force for dissociation (49.14 kcal/mol/nm), significantly higher than that for palbociclib (38.47 kcal/mol/nm). The higher unbinding force confirms that M24 interacts more tightly and persistently within the CDK4 active pocket. Collectively, these computational results suggest that M24 is the most promising 3-methyleneisoindolin-1-one derivative and may serve as a potential lead compound for the design of new CDK4 inhibitors with improved efficacy and reduced side effects compared to existing drugs [241].

**Figure 26:**
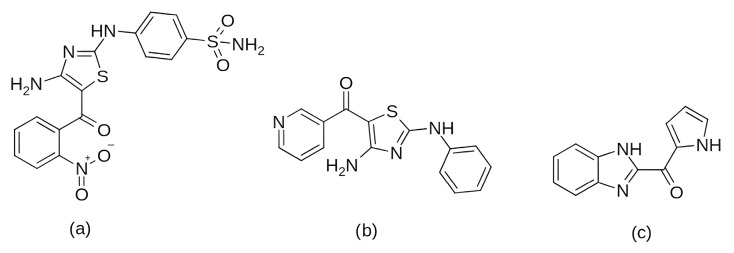
2D structures of the inhibitors X64 (**a**), X3A (**b**), and 4AU (**c**), as reported in the study by Wang et al. (2023) [242].

To further elucidate the molecular mechanisms underlying selectivity, Wang et al. (2023) [242] investigated the binding affinity of the inhibitors X64, X3A and 4AU (Figure 26) towards CDK2 and CDK6 with the aim of explaining differences in their selective activity. Three independent 400 ns MD simulations were performed in AMBER using the ff19SB force field for proteins, GAFF for inhibitors and the TIP3P water model. All three inhibitors formed stable complexes with CDK2 and CDK6, with RMSD values indicating conformational stability after approximately 100 ns. RMSF analysis revealed a higher structural flexibility of CDK6 compared to CDK2. Binding free energy analysis using the MM-GBSA method confirmed the higher affinity of all three inhibitors for CDK2 over CDK6, primarily due to more favorable van der Waals interactions and non-polar solvation free energy. A further decomposition of the energy contributions per residue revealed that differences between specific pairs of residues, such as (Ile18, Ile19), (Glu89, Arg90), (Phe90, Glu91), (Leu91, Thr92), and (Leu142, His143), played an important role in determining the different binding [242].

**Figure 27:**
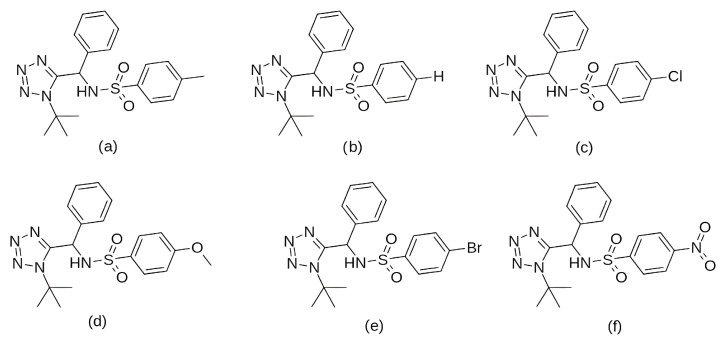
2D structures of the synthesized tetrazole derivatives DTS1 (**a**), DTS2 (**b**), DTS3 (**c**), DTS4 (**d**), DTS5 (**e**), and DTS6 (**f**), as reported in the study by Prabhu et al. (2024) [243].

Prabhu et al. (2024) [243] synthesized a series of six tetrazole derivatives (DTS1–DTS6; Figure 27). All compounds were confirmed as single stereoisomers with an R-configuration. Cytotoxicity assays revealed that these compounds were selectively active against ER-positive breast cancer cells, showing negligible effects on triple-negative MDA-MB-231 cells. Among them, DTS3, containing a chlorine substituent, exhibited the highest potency in inhibiting MCF-7 cell proliferation, with an IC_50_ value of approximately 200 μM after 48 h. Molecular docking, performed using the Glide module [244] in Maestro (Schrödinger), identified CDK6 as the most probable molecular target. DTS3 was predicted to bind stably within the active site of CDK6, forming hydrogen bonds and a specific halogen interaction between its chlorine atom and the Val101 residue, a known catalytic site. MD simulations under NPT conditions (300 K, 1.01325 bar) further supported the stability of the DTS3–CDK6 complex, showing four hydrogen bonds, RMSD values below 3 Å, and minimal fluctuations in Rg, indicating conformational stability. Collectively, these findings indicate that DTS3 is a promising anticancer agent with antiproliferative, apoptotic, and anti-invasive activity against ER-positive cancers, suggesting its possible role as a CDK6 inhibitor [243].

**Figure 28:**
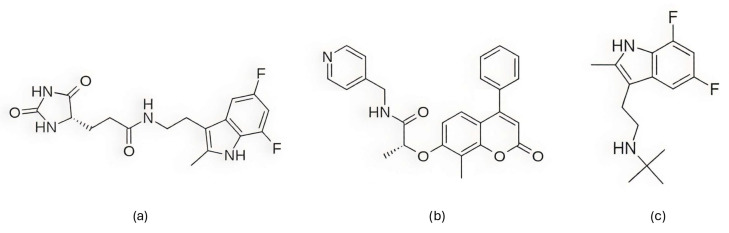
2D structures of compounds ZINC91325512 (**a**), ZINC04000264 (**b**), and R1 (**c**), identified as top inhibitors in the study by Dhiman et al. (2024) [245].

Using a pharmacophore-based approach, Dhiman et al. (2024) [245] performed a virtual screening of the ZINC database [246] to identify novel pyrrolo[2,3-d]pyrimidine (P2P) derivatives as potential CDK4/6 inhibitors. Among the top-scoring compounds obtained from XP docking in Glide (Schrödinger Maestro), ZINC91325512 (−9.171 kcal/mol) and ZINC04000264 (−9.134 kcal/mol) showed strong binding interactions with key amino acid residues: Ile19, Val101, and Lys147 in the case of ZINC91325512, and Ile19, Val101, and His100 for ZINC04000264. Both ligands displayed more favorable docking scores than the reference drug ribociclib (−8.548 kcal/mol). Their molecular structures are shown in Figure 28a,b. Guided by 3D-QSAR [247] contour maps, R-group enumeration led to the design of derivative R1 (Figure 28c), which formed hydrogen bonds with Val101 and Gln149, achieving an XP docking score of −8.931 kcal/mol—again outperforming ribociclib. To assess the structural stability of the ligand–protein complexes, 100 ns MD simulations were carried out in GROMACS using the OPLS3E force field, the SPC water model, and standard conditions (300 K, 1 bar). The average RMSD values indicated stable complexes for 5L2S-ZINC91325512 (1.733 Å), 5L2S-ZINC04000264 (1.610 Å), and 5L2S-R1 (1.649 Å), compared to 5L2S-ribociclib (2.298 Å). These lower RMSD values reflect reduced conformational fluctuations and stronger protein–ligand packing. RMSF and radius of gyration analyses further supported the rigidity and compactness of the complexes, identifying Val101 as a key residue contributing to stability through persistent hydrogen bonding and hydrophobic interactions. Binding free energy calculations using the MM/GBSA method indicate energetically favorable interactions for all three 5L2S–ligand complexes, with ZINC91325512 exhibiting the most negative ΔG (−61.79 kcal/mol), closely resembling that of abemaciclib (−62.79 kcal/mol) and more favorable than ribociclib (−53.67 kcal/mol). These findings suggest that ZINC91325512 and R1 form stable, thermodynamically favorable, and tightly bound complexes with CDK4/6, supporting their potential as lead scaffolds for further anticancer drug development [245].

**Figure 29:**
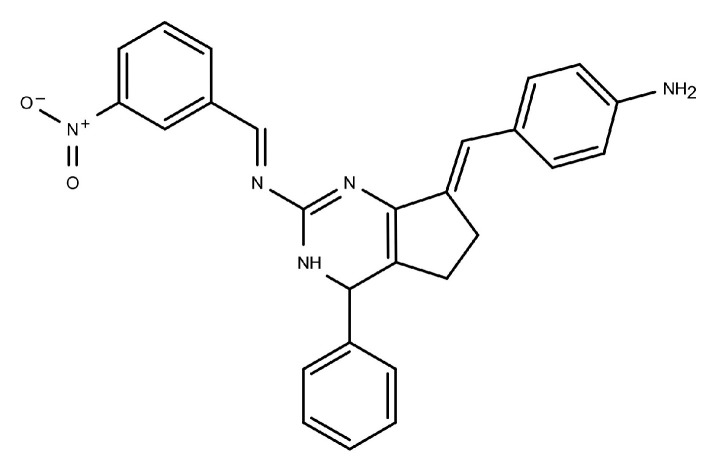
2D structure of the compound 2a3, as reported in the study by Manoharan et al. (2025) [248].

As a part of a rationally designed series of seventy-five derivatives based on the general N-(7-(substituted benzylidene)-4-phenyl-4,5,6,7-tetrahydro-3H-cyclopenta[d]pyrimidin-2-yl)-1-(substitutedphenyl)methanimine scaffold, Manoharan et al. (2025) [248] identified compound 2a3 (Figure 29) as a promising multitarget agent with high a binding affinity to several important regulatory proteins, including CDK4/6. Molecular docking, performed using AutoDock Vina-POAP [249], indicated that 2a3 binds strongly to p38 kinase (3LFF, −11.5 kcal/mol), FGFR (PDB ID: 5EW8 [250], −11.0 kcal/mol), MAPK (PDB ID: 6E2N [251], −10.3 kcal/mol), CDK4/6 (PDB ID: 6WW8 [252], −10.9 kcal/mol), HER2 (PDB ID: 7PCD [253], −11.1 kcal/mol), glyoxalase 1 (PDB ID: 7WT0 [254], −7.4 kcal/mol), and PI3K-α (PDB ID: 8EXL [255], −10.1 kcal/mol). Within the CDK4/6 binding site, 2a3 established multiple hydrogen-bond and hydrophobic interactions with residues Trp81, Pro82, Phe83, Gln85, Val87, Asp88, Lys91, Leu92, Leu94, Met132, Cys136 and, Tyr197 (Figure 2), stabilizing the complex. A 100 ns MD simulation (Desmond, Schrödinger) confirmed the structural stability of the 6WW8–2a3 complex, with RMSD values of 2.4 Å for the protein and 3.2 Å for the ligand, indicating strong conformational stability throughout the trajectory. Other protein–ligand systems remained comparatively stable, except for MAPK (6E2N), which showed greater fluctuation (24 Å protein; 48 Å ligand). Finally, MTT assay results on ER-positive MCF-7 cells confirmed potent cytotoxicity, with an IC_50_ value of 14.03 µM/mL, underscoring the potential of 2a3 as a stable, selective CDK4/6-centric anticancer lead [248].

## 7. Conclusions and Future Directions

CDK4/6 inhibitors have significantly improved clinical outcomes and have become a standard treatment strategy in both metastatic and high-risk early-stage HR+/HER2− breast cancer. However, the evidence reviewed here also shows that resistance and disease progression remain major clinical challenges, and that differences among the approved inhibitors are not fully explained by trial outcomes alone. Considered together, the clinical, structural, and computational data suggest that differences in selectivity, binding interactions, off-target activity, and physicochemical properties may contribute to differences in toxicity, tissue penetration, and resistance-related behavior. The main contribution of this review is therefore to show that the clinical behavior of CDK4/6 inhibitors can be understood more mechanistically when interpreted together with inhibitor binding and molecular mechanisms of resistance. Mechanisms such as RB1 inactivation, cyclin E/CDK2 activation, and alternative signaling pathways indicate that resistance is not only a clinical endpoint, but also a molecular process that may alter inhibitor sensitivity. At the same time, current evidence does not fully support predictive structure-based models of clinical response, and there is no evidence that personalized selection among the approved CDK4/6 inhibitors improves outcomes. Thus, linking structural differences to final therapeutic effect remains a promising but still unresolved challenge. Computational approaches are valuable because structural studies alone provide only a limited view of the dynamic and mechanistic features that influence inhibitor behavior. Docking, molecular dynamics simulations, and related in silico methods can help investigate conformational changes, interaction patterns, and the structural consequences of mutations, thereby supporting the interpretation of resistance mechanisms and the optimization of new agents. Future progress will depend on studies that more directly connect patient outcome, resistance biology, structural kinase features, and inhibitor properties.

## Figures and Tables

**Table 2:** Overview of the main clinical trials with CDK4/6 inhibitors, including patient population characteristics, treatment regimens, PFS and OS outcomes, and most common adverse effects. (Abbreviations: ABC, advanced breast cancer; aHR, adjusted HR; AI, aromatase inhibitor; CDK4/6, cyclin-dependent kinase 4 and cyclin-dependent kinase 6; CI, confidence interval; ET, endocrine therapy; HFS, hand and food syndrome; HR, hazard ratio; HR+/HER2−, hormone receptor-positive/human epidermal growth factor-negative; iDFS, invasive disease free survival; MBC, metastatic breast cancer; N/A, not available; NSAI, non-steroidal aromatase inhibitor; OS, overall survival; PFS, progression-free survival; TAM, tamoxifen; → followed by).

Clinical Trial		Phase	Population/Line of Treatment	Intervention	Number of Patients Evaluable	Median PFS or iDFS (Months)	Median OS (Months)	Adverse Effects (All Grades)
palbociclib	PALOMA-1/TRIO18 [122]	II	postmenopausal women with HR+/HER2− MBC, first line	palbociclib-letrozole vs. placebo-letrozole	165	20.2 vs. 10.2 (HR 0.49, 95% CI 0.32–0.75; *p* < 0.0001)	37.5 vs. 34.5 (HR 0.90, 95% CI 0.62–1.29; *p* = 0.28)	neutropenia 75% vs. 5%, fatigue 41% vs. 23%
	PALOMA-2/TRIO22 [123]	III	postmenopausal women with HR+/HER2− MBC, first line	palbociclib-letrozole vs. placebo-letrozole	666	27.6 vs. 14.5 (HR 0.56, 95% CI 0.46–0.69; *p* < 0.0001)	53.9 vs. 51.2 (HR 0.96, 95% CI 0.78–1.18; *p* = 0.34)	neutropenia 79.5% vs. 6.3%, fatigue 37.4% vs. 27.5%
	PALOMA-3 [124]	III	any menopausal status, HR+/HER2− MBC, after ET	palbociclib-fulvestrant vs. placebo-fulvestrant	521	9.5 vs. 4.6 (HR 0.46, 95% CI 0.36–0.59; *p* < 0.0001	34.8 vs. 28 (HR 0.81, 95% CI 0.65–0.99; *p* = 0.0414)	neutropenia 83% vs. 4%, anemia 30% vs. 13%
	PALOMA-4 [125]	III	any menopausal status, first-line therapy in Asian women with HR+/HER2− ABC	palbociclib-letrozole vs. placebo-letrozole	340	21.5 vs. 13.9 (HR 0.68, 95% CI 0.53–0.87; *p* = 0.0012)	N/A	N/A
	PEARL [126]	III	postmenopausal women with HR+/HER2− MBC resistant to previous AIs	capecitabine vs. palbociclib plus ET (exemestane, Cohort 1; fulvestrant, Cohort 2)	601	7.4 (palbociclib + fulvestrant) vs. 9.4 (capecitabine) (aHR 1.11, 95% CI 0.78–1.60; *p* = 0.54)	32.6 vs. 30.9 with palbociclib plus ET vs. capecitabine, respectively (aHR 1.00, 95% CI 0.82–1.23, *p* = 0.995)	(Available data only for grades 3–4); neutropenia: 58.5% vs. 5.9% (febrile: 1.0% vs. 1.4%); HFS: 0% vs. 24.2%; diarrhea: 1.3% vs. 7.6%; fatigue: 1.0% vs. 5.5%
	SONIA [127]	III	pre- and post-menopausal women with HR+, HER2− ABC	Strategy A: NSAI + CDK4/6 → fulvestrant upon progression vs. Strategy B: NSAI → fulvestrant with CDK4/6 inhibition upon progression	1050	31.0 cohort A vs. 26.8 cohort B (HR 0.87, 95% CI 0.74–1.03; *p* = 0.10)	24.7 cohort A vs. 16.1 cohort B (HR: 0.59, 95% CI 0.51–0.69; *p* < 0.0001)	N/A
	PALLAS [128]	III	patients with high-risk HR+/HER2− early-stage breast cancer	palbociclib for 2 years + ET vs. ET	5761	iDFS rates at year 4 84.2% vs. 84.5% (HR 0.96, 95% CI 0.81–1.14; *p* = 0.65)	At year 4: 93.8% vs. 95.2% (HR, 1.32 (95% CI, 0.98 to 1.78, *p* = 0.71)	neutropenia: 83.5% vs. 4.9%, fatigue: 41% vs. 19.3%, anemia 23.6% vs. 5.4%, thrombocytopenia 21.7% vs. 1.7%
	PALMIRA [129]	II	patients with HR+/HER2−, after progression on first-line palbociclib	palbociclib + ET vs. ET	198	4.9 vs. 3.6 (HR 0.84, 95% CI 0.66–1.07; *p* = 0.149	28.0 vs. 28.8 (HR 0.84, 95% CI 0.66–1.07; *p* = 0.149)	neutropenia 52.6% vs. 1.7%, anemia 18.5% vs. 8.3%, fatigue 27.4% vs. 23.3%, nausea 11.9% vs. 11.7%
ribociclib	MONALEESA-2 [130]	III	patients with HR+/HER2− recurrent/MBC, first line	ribociclib-letrozole vs. placebo-letrozole	668	25.3 vs. 16.0 (HR 0.57, 95% CI 0.46–0.70; *p* < 0.001)	63.9 vs. 51.4 (HR 0.76, 95% CI 0.63–0.93; *p* = 0.008)	neutropenia 74.3% vs. 5.2%, anemia 57% vs. 4%, nausea 51.5% vs. 28.5%, liver toxicity 46% vs. 44%, prolonged QTc (only grade 3 and 4 are reported) 4.5% vs. 2.2%
	MONALEESA-3 [131]	III	patients with HR+/HER2− ABC, first or second line	ribociclib-fulvestrant vs. placebo-fulvestrant	484	20.5 vs. 12.8 (HR 0.59, 95% CI 0.48–0.73; *p* < 0.001)	53.7 vs. 41.5 (HR 0.73, 95% CI 0.59–0.90)	neutropenia 69.6% vs. 2.1%, anemia 17% vs. 5%, thrombocytopenia 33% vs. 11%, nausea 45.3% vs. 28.2%, fatigue 31% vs. 33%
	MONALEESA-7 [132]	III	premenopausal women with HR+/HER2− ABC, no previous CDK4/6, allowed neo/adjuvant or up to one line of chemotherapy	ribociclib-ET vs. placebo-ET	672	23.8 vs. 13 (HR 0.55, 95% CI 0.44–0.69; *p* < 0.0001)	58.7 vs. 48 (HR 0.76, 95% CI 0.61–0.96)	neutropenia 76% vs. 8%, nausea 32% vs. 19%, liver toxicity 12% vs. 7%, QTc prolongation 16.1 (with TAM) and 7.3 (with NSAI) vs. 0
	NATALEE [133,134]	III	patients with high-risk early-stage breast cancer	ribociclib for 3 years + ET vs. placebo for 3 years + ET	5101	iDFS at year 4: 88.5% vs. 83.6% (HR 0.72, 95% CI 0.61–0.84; *p* < 0.01)	N/A	neutropenia 62.8% vs. 4.5%, thrombocytopenia 28% vs. 13%, anemia 47% vs. 26%, nausea 23.5% vs. 7.9%, fatigue 22.8% vs. 13.5%, liver toxicity 45% vs. 35%, QTc prolongation 4.3% vs. unknown for the control arm
	MAINTAIN [135]	II	patients with HR+/HER2− metastatic disease after progression on palbociclib + ET	ribociclib + switched ET vs. placebo + switched ET	119	5.29 vs. 2.76 (HR 0.57, 95% CI 0.39–0.85; *p* = 0.006	N/A	neutropenia 72% vs. 15%, thrombocytopenia 25% vs. 22%, anemia 23% vs. 22%
abemaciclib	MONARCH-2 [136]	III	pre- and post-menopausal women with HR+/HER2− ABC progressed while receiving ET (neo/adjuvant of first line)	abemaciclib-fulvestrant vs. placebo-fulvestrant	669	16.4 vs. 9.3 (HR 0.55, 95% CI 0.45–0.68, *p* < 0.001)	46.7 vs. 37.2(HR 0.78, 95% CI 0.64–0.96; *p* = 0.01)	diarrhea 86.4% vs. 24.7%, neutropenia 46.0% vs. 4.0%, nausea 45.1% vs. 22.9%
	MONARCH-3 [36]	III	postmenopausal HR+/HER2− ABC, first line	abemaciclib-NSAI vs. placebo-NSAI	493	28.2 vs. 14.8 (HR 0.54, 95% CI 0.42–0.70)	67.1 vs. 53.7 (HR 0.804, 95% CI 0.64–1.02; *p* = 0.067)	diarrhea 81.3% vs. 29.8%, neutropenia 41.3% vs. 1.9%, fatigue 40.1% vs. 31.7%
	MONARCH-E [137,138]	III	high-risk early-stage HR+/HER2− breast cancer	abemaciclib-ET vs. ET alone	5637	4-year iDFS 85.8% vs. 79.4% (HR 0.66, 95% CI 0.58–0.76; *p* < 0.001)	N/A	diarrhea 84% vs. 9%, nausea 20% vs. 9%, fatigue 41% vs. 18%, neutropenia 84 vs. 23%, thrombocytopenia 37% vs. 10%, anemia 68% vs. 17%

**Table 3:** Selective CDK4/6 inhibitors currently approved and in development for the treatment of breast cancer.

Drug Name	Developer	Stage	IC_50_
palbociclib (PD-0332991) [143,144]	Pfizer	approved for HR+/HER2− metastatic breast cancer	CDK4: 11 nM CDK6: 16 nM
ribociclib (LEE011) [143,144]	Novartis	approved for HR+/HER2− metastatic breast cancer, and for high-risk early-stage breast cancer	CDK4: 10 nM CDK6: 39 nM
abemaciclib (LY-2835219) [143,144]	Eli Lilly	approved for HR+/HER2− metastatic breast cancer, and for high-risk early-stage breast cancer	CDK4: 2 nM CDK6: 10 nM CDK9: 57 nM
dalpiciclib (SHR6390) [145]	Jiangsu Hengrui Medicine	phase III	CDK4: 12 nM CDK6: 10 nM
lerociclib [143,146]	G1 Therapeutics	phase I/II	CDK4: 1 nM CDK6: 2 nM CDK9: 28 nM.
tibremciclib (BPI-16350) [143]	Betta Pharmaceuticals	phase III	*N/A
TQB3616 [147]	CTTQ Pharma	phase I	N/A
FCN-437 [146]	Ahon Pharmaceutical	phase III	N/A
birociclib (XZP-3287) [143,146]	Sihuan Pharmaceutical/XuanZhu Pharma	phase II	N/A
HS-10342 [143,144]	Jiangsu Hansoh Pharmaceutical Group	phase II	N/A
CS3002 [143]	CStone Pharmaceuticals	phase I	N/A
BEBT-209 [143]	Guangzhou BeBetter Medicine Technology	phase I	N/A
BPI-1178 [146]	Beta	phase I/II	N/A
PF-06873600 [148]	Pfizer	phase II	CDK4: 0.13 nM Ki CDK6: 0.16 nM Ki
TY-302 [146]	TYK Medicines	phase II	N/A
PD0183812 [149]	Pfizer	phase I	CDK4: 8 nM CDK6: 13 nM
SRX3177 [150]	G1 Therapeutics	preclinical phase	CDK4: 2.5 nM CDK6: 3.3 nM

*N/A—not available.

## Data Availability

No new data were created or analyzed in this study.

## Supplementary Materials

The following supporting information can be downloaded at: https://www.mdpi.com/article/10.3390/ph19040610/s1, Figure S1: Superposition of CDK2 in the inactive T-loop-in conformation (1HCK) (gray) and the more active-like T-loop-out conformation (1JST) (blue). The kinase backbones are shown as cartoons in pale colors, and the T-loop regions are highlighted in nontransparent colors. The marked displacement of the T-loop illustrates the conformational transition associated with CDK2 activation and provides a structural framework for discussing conformation-dependent inhibitor recognition.

## Abbreviations

The following abbreviations are used in this manuscript:

ABC	Advanced breast cancer
AE	Adverse effect
AI	Aromatase inhibitor
ALT	Alanine transaminase
AMBER	Assisted Model Building with Energy Refinement
APE	Ala-Pro-Glu
ASK1	Apoptosis signal-regulating kinase 1
AST	Aspartate transaminase
ATP	Adenosine triphosphate
BC	Breast cancer
CAK	CDK-activating kinase
CDK	Cyclin-dependent kinase
CDK4/6	Cyclin-dependent kinases 4 and 6
CHARMM	Chemistry at HARvard Macromolecular Mechanics
CKI	Cyclin-dependent kinase inhibitor
C_max_	Maximum plasma concentration
CMNPD	Comprehensive Marine Natural Products Database
CYP3A4	Cytochrome P450 3A4
DFG	Asp-Phe-Gly
DNA	Deoxyribonucleic acid
EGCG	Epigallocatechin gallate
ER+	Estrogen receptor positive
ET	Endocrine therapy
FDA	Food and Drug Administration
FGFR	Fibroblast growth factor receptor
GROMACS	GROningen Machine for Chemical Simulations
GROMOS	GROningen Molecular Simulation
HER2	Human epidermal growth factor receptor 2
HFS	Hand-foot syndrome
HR+	Hormone receptor positive
IC_50_	Half-maximal inhibitory concentration
IDFS	Invasive disease-free survival
IFD	Induced Fit Docking
Ka	Association constant
KID	Kinase inhibitory domain
LC-MS/MS	Liquid Chromatography–Tandem Mass Spectrometry
MAPK	Mitogen-activated protein kinase
MBC	Metastatic breast cancer
MCF-7	Michigan Cancer Foundation-7
MD	Molecular dynamics
MM-GBSA	Molecular Mechanics Generalized Born Surface Area
MM-PBSA	Molecular Mechanics Poisson–Boltzmann Surface Area
MNP	Marine Natural Product
NAMD	Nanoscale molecular dynamics
NSAI	Non-steroidal aromatase inhibitor
ONIOM	Our Own N-layered Integrated molecular Orbital and Molecular mechanic
OPLS	Optimized Potentials for Liquid Simulations
OS	Overall survival
PDB	Protein Data Bank
PFS	Progression-free survival
PiKα	Phosphatidylinositol 3-kinase alpha
pLDDT	Predicted Local Distance Difference Test
Rb	Retinoblastoma protein
RCSB	Search Collaboratory for Structural Bioinformatic
Rg	Radius of gyration
RMSD	Root-mean-square deviation
RMSF	Root-mean-square fluctuation
ROCs	Receiver operating characteristics
SASA	Solvent-accessible surface area
See-SAR	See Structure–Activity Relationships
SNP	Single-nucleotide polymorphism
TAM	Tamoxifen
TNBC	Triple-negative breast cancer
YASARA	Yet Another Scientific Artificial Reality Application
