# Supplementary material for: CDK4/6 Inhibitors for Breast Cancer Therapy—A Review of Clinical Trials, Structural and Computational Approaches
Source: Pharmaceuticals (Basel). 2026 Apr 10;19(4):610. doi: 10.3390/ph19040610 (PMC13118760; doi:10.3390/ph19040610)
Supplement: Supplementary file 1 [file pharmaceuticals-19-00610-s001.zip › pharmaceuticals-4215212-supplementary.pdf]

CDK4/6 Inhibitors for Breast Cancer Therapy - A Review of Clinical Trials, Structural and Computational Approaches

Adela Avdičević [1], Samo Lešnik [1,2], Urban Bren [1,2,3], Luka Čavka [4,*]

Correspondence: luka.cavka@ukc-mb.si

Supplementary materials

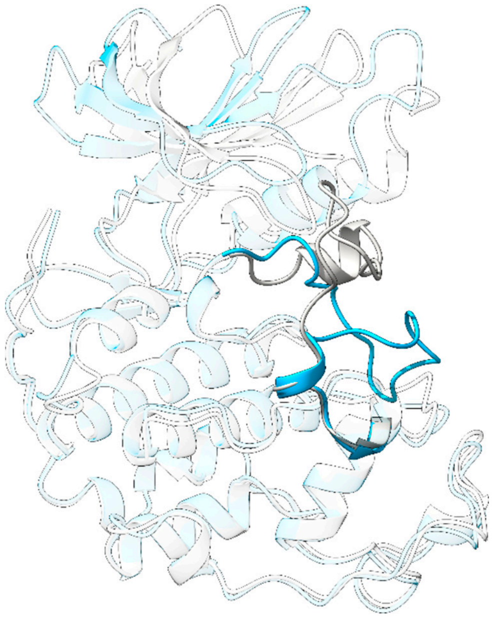

Figure S1. Superposition of CDK2 in the inactive T-loop-in conformation (1HCK) (gray) and the more active-like T-loop-out conformation (1JST) (blue). The kinase backbones are shown as cartoons in pale colors, and the T-loop regions are highlighted in nontransparent colors. The marked displacement of the T-loop illustrates the conformational transition associated with CDK2 activation and provides a structural framework for discussing conformation-dependent inhibitor recognition.
